# A Review on Bio- and Chemosensors for the Detection of Biogenic Amines in Food Safety Applications: The Status in 2022

**DOI:** 10.3390/s23020613

**Published:** 2023-01-05

**Authors:** Stella Givanoudi, Marc Heyndrickx, Tom Depuydt, Mehran Khorshid, Johan Robbens, Patrick Wagner

**Affiliations:** 1 Technology and Food Science Unit, Flanders Research Institute for Agriculture, Fisheries and Food (ILVO), Brusselsesteenweg 370, B-9090 Melle, Belgium; 2 Laboratory for Soft Matter and Biophysics, ZMB, Department of Physics and Astronomy, KU Leuven, Celestijnenlaan 200 D, B-3001 Leuven, Belgium; 3 Animal Sciences Unit, Flanders Research Institute for Agriculture, Fisheries and Food (ILVO), Marine Division—Cell Blue Biotech/Food Integrity, Jacobsenstraat 1, B-8400 Oostende, Belgium

**Keywords:** biogenic amines, food safety, bio- and chemosensors, biomimetic receptors, aptamers, molecularly imprinted polymers, antibodies, enzymes

## Abstract

This article provides an overview on the broad topic of biogenic amines (BAs) that are a persistent concern in the context of food quality and safety. They emerge mainly from the decomposition of amino acids in protein-rich food due to enzymes excreted by pathogenic bacteria that infect food under inappropriate storage conditions. While there are food authority regulations on the maximum allowed amounts of, e.g., histamine in fish, sensitive individuals can still suffer from medical conditions triggered by biogenic amines, and mass outbreaks of scombroid poisoning are reported regularly. We review first the classical techniques used for selective BA detection and quantification in analytical laboratories and focus then on sensor-based solutions aiming at on-site BA detection throughout the food chain. There are receptor-free chemosensors for BA detection and a vastly growing range of bio- and biomimetic sensors that employ receptors to enable selective molecular recognition. Regarding the receptors, we address enzymes, antibodies, molecularly imprinted polymers (MIPs), and aptamers as the most recent class of BA receptors. Furthermore, we address the underlying transducer technologies, including optical, electrochemical, mass-sensitive, and thermal-based sensing principles. The review concludes with an assessment on the persistent limitations of BA sensors, a technological forecast, and thoughts on short-term solutions.

## 1. Introduction

### 1.1. Biogenic Amines from a Biochemical Perspective

**Figure 1:**
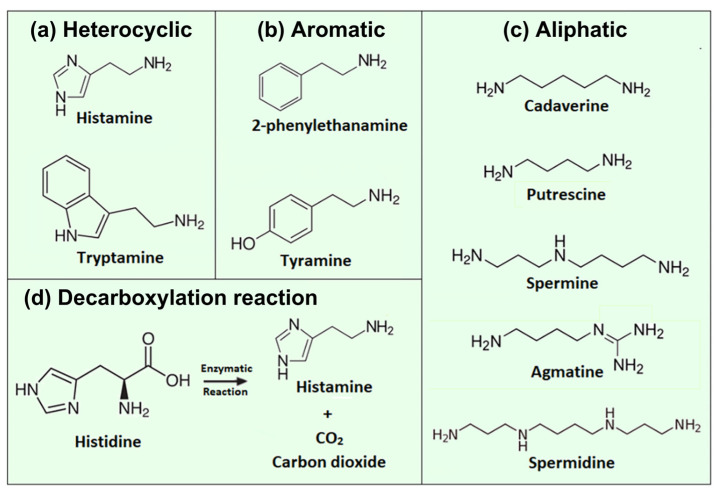
Chemical structures of important biogenic amines playing a role in food safety, including (**a**) heterocyclic, (**b**) aromatic, and (**c**) aliphatic examples. Panel (**d**) shows schematically the enzymatic decomposition of its parent compound, the amino acid histidine, into histamine and carbon dioxide. Due to its structural similarity, histidine is frequently used as a competitor molecule to assess the selectivity of natural and synthetic histamine receptors.

Biogenic amines (BAs) are nitrogenous compounds synthesized enzymatically by various living organisms through the decarboxylation of amino acids. Based on the number of amino groups in their structure, BAs can be classified as monoamines (which include compounds with a single amine group) and polyamines, containing multiple amine groups. Structurally, biogenic amines are heterocyclic, aromatic, or aliphatic compounds as illustrated in Figure 1. The names of biogenic-amine molecules often reflect their amino-acid precursors: Histamine emerges from histidine, tryptamine from tryptophan, 2-phenylethanamine from L-phenylalanine, and tyramine from tyrosine. Cadaverine is, produced from lysine, while agmatine stems from arginine; consecutively, agmatine transforms enzymatically to putrescine, spermine, and spermidine, all with an unpleasant, ammonia-like odor. 

Biogenic amines play an important role in a large number of biological processes. In prokaryotic microorganisms, biogenic amines contribute to the survival of bacteria under environmental stress [1,2] and in some species they also participate in the generation of metabolic energy through the decarboxylation reaction [3]. In animals, biogenic amines play a role in various biological processes, from cell growth and differentiation to neural transmission. Histamine is naturally produced and released upon stimulation from human basophils, mast cells, and enterochromaffin-like cells [4,5]. Histamine is the ligand of the H1, H2, H3, and H4-type receptors, which are located in a variety of cells throughout the body [6]. Histamine receptors belong to the G-protein-coupled family of receptors and, upon activation by histamine, they provoke a variety of physiological effects, such as inflammation, gastric acid secretion and maintenance of the circadian rhythm. In the brain, histamine functions as a neurotransmitter, and it is involved in the sense of appetite, the state of wakefulness/sleepiness, and memory [7].

### 1.2. Occurrence of BAs in Food and Beverages

Despite the fact that BAs are naturally produced by living organisms, they can be hazardous when they accumulate in high levels. An increased dietary intake of BAs can lead to adverse health effects, which are addressed in Section 1.3. The BAs that are most commonly found in food include histamine, tyramine, agmatine, spermine, spermidine, cadaverine, and putrescine. BAs are naturally present in food, but their amounts can increase significantly due to the presence of decarboxylase-producing microorganisms. For this reason, BAs are considered as indicators for microbial food contamination. Microorganisms can be present in food products intentionally for fermentation purposes or due to unwanted spoilage: Bacteria, such as Lactobacillales, are extensively used in the production of fermented food and beverages including meat, dairy products, wine, and beer. Other histamine-producing bacteria have been isolated from fish [8,9,10,11,12], cheese [13], and wine [14,15]. Strongly histamine-producing bacterial species that were found in various marine fishes and seafood are *Morganella morganii*, *Klebsiella oxytoca*, *Proteus vulgaris*, *Proteus mirabilis*, *Providencia stuartii*, *Providencia rustigianii*, *Citrobacter braakii*, and *Serratia fonticola*, and a complete list is provided in ref. [16]. Histamine-forming bacteria can be found, especially in the skin, gills, and the intestines of these fishes [9].

Among the histamine-producing lactic-acid bacteria, there are species that are used as starter cultures for fermented milk products and wines. For example, strains of *Lactococcus lactis*, *Lactobacillus helveticus*, *Oenococcus oeni*, and *Leuconostoc mesenteroides* are used to produce fermented vegetables (e.g., gherkins, pickles, sauerkraut, and kimchi); dairy (e.g., yoghurt, cheese, kefir, kvass, and kombucha); several meat products; and for wine production [17]. Other bacteria, more specifically strains of *Enterococcus faecium*, *Lactobacillus brevis*, and *L. hilgardii* that were isolated from wine, are known to produce tyramine and have tyrosine decarboxylase activity [18,19]. Moreover, strong tyramine-producing strains of *Lactococcus* and *Leuconostoc* have been isolated from milk [20]. Despite the fact that such strains are not used intentionally for fermentation processes, they are considered as spoilage microflora of the manufacturing environment, and they can potentially contaminate the food products. The ability of BA-producing bacteria to form biofilms has been shown for polystyrene- and stainless-steel surfaces that are used extensively in food industry [21,22]. It is noteworthy that there are also numerous bacterial strains, that produce amine-degrading enzymes, offering potentially a way to reduce the BA load in food products (see the recent review by Li and Lu in ref. [23]).

**Figure 2:**
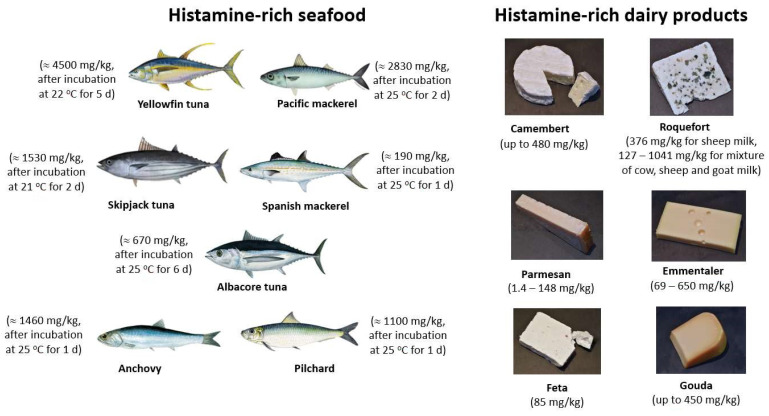
Seafood and dairy products containing ca. 100 mg histamine or more per kilogram. The given concentrations are either average values or indicate the minimum-maximum concentration range in mg/kg. In the case of fish, all samples were stored for at least one day at room temperature or slightly above. The histamine contents are based on the references [25,26,34]. The fish images are adopted from ref. [35], courtesy of NOAA Fisheries; the photographs of cheese types were provided by © Hans Hillewaert, ILVO.

Among the BAs, histamine, cadaverine, and putrescine are commonly present in fish products, and they are considered as indicators of seafood freshness and quality. Their presence in high amounts indicates food spoilage by microbial contamination, which can result from poor manufacturing practices and incorrect storage during processing of seafood. Together with tyramine and tryptamine, histamine, cadaverine, and putrescine are also present in dairy and meat products. The BA contents of various foodstuff (vegetables, fish, dairy products, fermented meat products, and beverages) have been reviewed extensively [24,25,26,27,28,29,30]. Figure 2 gives an overview on selected dairy products and fish species that all have histamine concentrations exceeding 100 mg/kg, which can even rise to 4500 mg/kg in Yellowfin tuna after storing it on purpose for 5 days at 22 °C. Most of the histamine-rich fish species belong zoologically to the families of *Scombroidae* (scombroids) and *Scomberesocidae*, lending their name to the related foodborne illness scombroid food poisoning SFP [31]. Interestingly, several BAs have been detected also in alcohol-free, non-fermented beverages such as plant milks (e.g., soymilk and oat milk), fruit juices, tea, and coffee; the concentrations in most of these examples are typically below 10 mg/kg, most often even blow 1 mg/kg [32]. The topic of BA detection in dairy products, including the required sample-pretreatment methods, is covered in detail in a review of 2022 by Moniente and coworkers [33].

### 1.3. Adverse Health Effects Due to Intake of BAs

Scombroid food poisoning results in extensive redness of the skin, respiratory problems, tachycardia, burning sensations in the throat, dizziness, headache, and nausea [36]. In many cases, SFP causes only mild symptoms but, in exceptional situations, SFP can affect the breathing severely and lead to life-threatening complications [37,38]. Often, it is misdiagnosed as an allergic reaction to fish, because it shares similar symptoms with immunoglobulin (IgE)-mediated allergies [31]. It is also known from the literature that SFP symptoms can be mistaken for salmonellosis [39]. Evidently, misdiagnosis leads to under-reporting of SFP cases among the food poisoning incidents. Although SFP is mainly related to seafood poisoning, it was also reported after consumption of contaminated cheese [40]. Symptoms can last from a few hours up to a maximum of 36 h and a correct clinical diagnosis requires measuring the histamine level in the blood plasma, and the level of histamine metabolites in the urine of the patient [41]. Treatment, if necessary, involves fast-acting antihistamines and supportive care. 

High-risk fishes for SFP are the histidine-rich ones such as tuna, anchovies, sardines, mackerel, mahi-mahi and herring (see Figure 2). When stored properly, these fishes are safe for consumption. However, keeping them for 24 h at 20 °C leads to histamine levels that are no more on the safe side [42]. The decomposition of histidine to histamine by the microbial enzymes in fish occurs most efficiently at a temperature of 25 °C, which holds also for fish that had previously been frozen [43]. At lower temperatures (8 °C), the formation of histamine in fish is delayed, but it can still reach critical levels after 4 days of storage [10,42]. It is worthwhile to mention that fish with high concentrations of histamine may still retain a normal, unsuspicious appearance and odor [44], but it is unsafe for consumption. Furthermore, histamine is heat-stable and will not be inactivated by elevated temperatures such as during cooking and smoking [41].

While intake of histamine-contaminated seafood is the main reason for SPF [41], it is also known that oral intake of pure histamine by healthy volunteers causes only mild SFP symptoms [45]. The major enzymes responsible for the metabolic degradation of histamine are diamine oxidase DAO and histamine-N-methyltransferase HNMT [46]. Both enzymes are expressed in a variety of human tissues and most importantly in the intestinal epithelium. Hence, this enzymatic activity prevents that too high amounts of histamine enter the blood stream [47,48]. Moreover, it was shown that 80% of histamine that still passes the intestinal barrier is metabolized in the blood and discarded via the kidney within 24 h [49]. Thus, the fast degradation of histamine in the body has a protective role against histamine intoxication. 

A predisposition to histamine intoxication can have several reasons, including the insufficient catabolism in the human body: Low levels of enzymatic activity of DAO and HNMT can lead to an impaired degradation and increased accumulation of histamine in the blood. Several genetic and environmental factors can contribute to insufficient activity of both enzymes [46]. The most important environmental factor are foodborne inhibitors belonging to the mono- and polyamines. For example, tyramine, tryptamine, 1-phenylethylamine, and agmatine inhibit HNMT, while agmatine and tyramine act in addition also as inhibitors for DAO [50]. Such mixtures of BAs are more likely to be present in complex food samples than in a controlled experiment with chemically pure histamine [44]. Two other biogenic amines, cadaverine and putrescine (also found in seafood) are known to enhance the toxicity of histamine and to contribute to the onset of SFP [49,51,52,53]. According to experiments on rats, cadaverine is a strong inhibitor of DAO and a weak inhibitor of HNMT [50]. Moreover, cadaverine and putrescine are known as precursors for carcinogenic nitrosamines [54]. 

Regarding other BAs, spermine and spermidine are nutrients that play multiple physiological roles in cells, ranging from gene expression to cell proliferation [55]. Notwithstanding, the accumulation of spermine and spermidine in the organism can reverse their benefits and even lead to nephrotoxic and spermicidal effects [56]. In rats, spermine and spermidine cause acute toxicity already at 600 mg per kg of body weight, which is the lowest toxic concentration threshold among all dietary biogenic amines [57]. Tyramine and tryptamine are biogenic amines that contribute, for instance, to neurotransmission. In high amounts however, they can be a health risk for consumers, particularly for patients using monoamine–oxidase-inhibiting drugs that are prescribed for psychiatric and neurological disorders [58]. Especially ripened cheeses can be rich in tyramine and concentrations above 1080 mg per kg food product are considered as toxic [59,60]. The illness resulting from tyramine intake is known as “cheese effect” with headache, vomiting, perspiration, and hypertensive crises as typical and potentially severe symptoms [61,62]. Already low amounts of tyramine can cause this effect in individuals when genetic or environmental factors (e.g., MAOI-type antidepressants) and reduce the enzymatic activity of MAO [28]. 

### 1.4. Legal Regulations on the BA Contents in Food Products

It is evident that biogenic amines should must not accumulate in high concentrations in food products due to their hazardous effects on human health. In experiments on rats, Til and coworkers have determined the median lethal dose (LD50) for cadaverine that is 5000 mg per kg of body weight and 2000 mg/kg both for putrescine and tyramine [57]. A corresponding LD50 dose for histamine could not be found in the literature. Despite of this, the legislation around biogenic amines focuses mainly on the limits of histamine in fish products. Regarding other food products and other biogenic amines, no specific limits have been set so far. The level of histamine in fish products is strictly regulated within the European Union and the United States of America [63,64]. According to the European Food Safety Authority (EFSA, Panel on Biological Hazards 2011), histamine is considered safe for consumption at 50 mg of histamine per meal for healthy individuals; however, the tolerable amount drops significantly for patients with histamine intolerance [65]. This condition is characterized by increased histamine absorption through the intestines and a reduced metabolic histamine degradation, leading to the accumulation of histamine in the blood. For tyramine, the NOAEL (no-observed-adverse-effect level) is set at 200 mg of tyramine per meal for healthy persons and as low as 5 mg for patients under MAO-inhibiting therapeutic treatments [66]. Within the European Union, a food safety criterion for histidine-rich fish and enzyme-treated histidine-rich fish products has been defined (see also ref. [67] for more details). The legislation is based on measuring an average of nine samples per batch: In the case of fish, at least seven samples must not exceed a limit of 100 mg/kg while, at maximum, two samples may contain at most 200 mg/kg. For enzyme-treated fish products, at least seven samples must stay below 200 mg/kg and a maximal of two samples may have histamine concentrations up to 400 mg/kg. In the USA, the critical limit for histamine in fish has been set slightly lower at 50 mg/kg [64]. No legal limits exist for cadaverine and putrescine until date.

Larger outbreaks of histamine poisoning have been reported in various countries, including the United States [68,69,70], Australia [71], Israel [72], Senegal [73], Peru [74], and Taiwan [75,76]. This list is not exhaustive, and minor outbreaks are often not documented in the scientific literature. A comparative study on fish and fish products purchased in nine different countries reveals that fish from certain origins is more likely to contain elevated histamine levels [77]. However, also in the EU, samples were identified that did not fulfill the legal limits. One should be aware that the globalization of the seafood market and lack of uniform regulations increase the necessity of monitoring the histamine level in fish and its derived products, ideally at the country of origin, as well as the country of consumption.

## 2. Sensor-Based Techniques for BA Detection

### 2.1. Analytical Reference Methods for BA Quantification and Identification

Up to now, the identification and quantification of biogenic amines is mostly based on chromatographic techniques: These techniques include thin layer chromatography (TLC), gas chromatography (GC), reversed-phase high-performance liquid chromatography (RP-HPLC), ion chromatography, and capillary electrophoresis (CE). The official reference method for routine testing of fish products for compliance with the food safety criteria is HPLC [67]. Chromatographic techniques require extensive sample preparation for the extraction of biogenic amines from their solid or aqueous matrices. These include solid-phase extraction, matrix solid-phase dispersion and column-based separation of the biogenic amines. It is beyond the scope of this review to give details about the chromatography-based identification of biogenic amines, the reader may refer to several overview articles (see [78,79,80,81,82,83]). 

The aim of the present review is to provide a comprehensive overview on the field of bio- and chemosensors for the detection of biogenic amines in a food safety perspective. These sensors are generally compact devices, holding the promise to enable fast and facile BA detection and quantification in an on-site setting, outside an analytical laboratory. Histamine detection is an especially active field, but we will also address other biogenic amines that are important in terms of food safety. There are several other review articles available on BA detection: The works by Kivirand and Rinken, as well as by Verma et al., focus on biosensors based on enzymatic reactions [84,85]. The review by Vasconcelos and coworkers includes again enzymes but considers also aptamers and molecularly imprinted polymers as receptors [86]. A recent article by Gomes Müller et al. discusses classical analytical techniques (mass spectrometry and chromatography) together with various optical- and electrochemical methods [87]. A dedicated review on optical sensors for BA detection was recently published by Danchuk and coworkers [88]. Our article is structured differently and follows the line of the receptor technology, including also receptor-free sensors and commercially available fast tests.

### 2.2. Receptor-Free Chemosensors for Biogenic Amines

The detection of BAs is possible without using biorecognition elements, provided that the BAs can be changed chemically into detectable products. Such sensors employ electroanalytical methods with potentiometry and voltammetry as important examples. For convenience, we refer to these sensors as nBRE sensors (no biorecognition element sensors). The electroactive biogenic amines can be oxidized reversibly or irreversibly and the oxides, which are formed on the surface of the sensor, are detected with electrochemical transducers. The analytical signal, recorded as changes in the potential or current, reflects the concentration of BAs. Various strategies have been developed for the electrochemical detection of BAs. For example, histamine was detected by cyclic voltammetry through its direct electro-oxidation at a carbon nanotube-modified electrode [89]. Young et al. fabricated a thin film of pyrroloquinoline quinone (PQQ) on a glassy carbon electrode for amperometric histamine detection [90]. The proposed detection mechanism is based on the nonenzymatic oxidative deamination of amines by PQQ, an electron transfer mediator. Studies were conducted to understand the electrochemical properties of both soluble and immobilized PQQ at electrode surfaces [91,92]. PQQ is a redox coenzyme of the PQQ-dependent oxidoreductases (quinoproteins) that undergoes cycles of reduction and regeneration with the transfer of two electrons and protons [93]. Even in nonenzymatic systems, PQQ catalyzes oxidation reduction cycles, resulting in the oxidation of substrates [94,95]. Primary amine-containing compounds, such as BAs, can be oxidized by PQQ [96]. In ref. [97], the authors applied square wave stripping voltammetry using a hanging mercury drop electrode to determine histamine in the presence of copper (Cu). Their method was based on the complexation of histamine with transition metals such as Cu, Ni and Pd [98]. The reduction signal of the histamine–Cu complex was observed to be strongly pH-dependent. Since the pH affects the electrochemical signal of histamine oxidation, the pH needs to be carefully adjusted [90,99,100,101]. 

**Figure 3:**
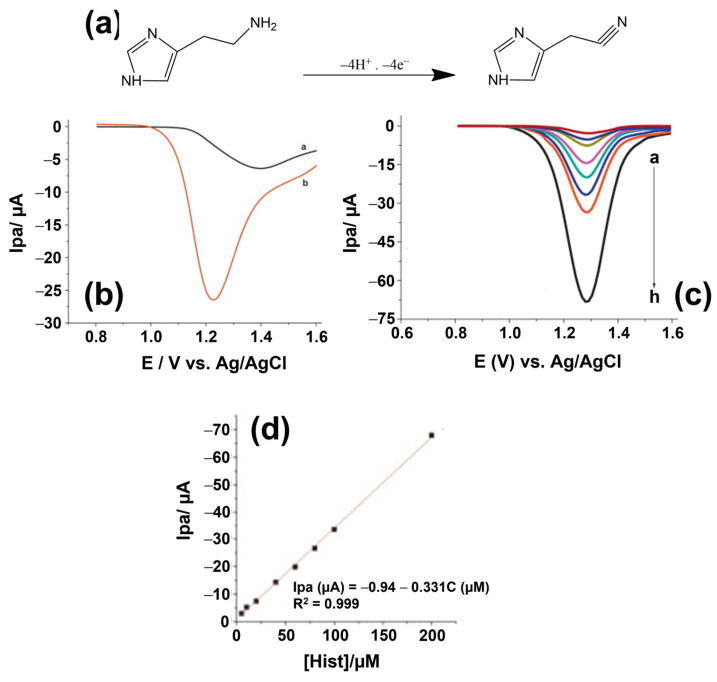
(**a**) Electrochemical reaction scheme for the oxidation of histamine with one possible pathway, at least four different oxidation products of histamine are known in the literature [94,99,103]. (**b)** Applying square-wave voltammetry, the peak current of a lignin-modified glassy carbon electrode (GCE, curve a) is four times higher than for a non-modified GCE, while the overpotential is reduced by 180 mV (curve b) for 1 mM histamine. (**c**) Square-wave voltammograms for increasing histamine concentrations from 5 μM (curve a) to 200 μM (curve h). (**d)** Plotting the peak currents *Ipa* of panel (**c**) as a function of the histamine concentration reveals a broad linear range from 5 to 200 μM. The figure is reprinted and modified from ref. [100] by Degefu et al., Copyright 2014, with permission from Elsevier.

Electrodes that have been used for the detection of biogenic amines include boron- doped diamond [102], metal or glassy carbon electrodes [103] and gold electrodes [104]. The surface of the electrode can be modified with various modifiers including carbon nanotubes [89,99], gold nanoparticles [105], conducting polymers such as lignin and poly(4-amino-3-hydroxynaphthalene sulfonic acid (p-AHNSA) [99,100], nickel [103], and thin mercury films [106]. An illustration of the improved sensing performance of lignin-coated glassy carbon electrodes can be seen in Figure 3. These modifiers, all used in nBRE-type BA sensors, improve the stability, increase the active surface area, and the electroactive properties of the electrode. For example, nanocarbons exhibit high electrical conductivity and enhance the electrochemical signal, because they facilitate the electron transfer between the biogenic amines and the electrode surface [107]. Furthermore, modifiers that create a negative surface charge at an electrode will attract positively charged, protonated BA molecules by electrostatic interactions. In turn, the enhanced BA concentration close to the electrode surface will increase voltametric and amperometric signals. In another example, Amorim et al. synthesized an electrode modified with a polymeric membrane for the potentiometric detection of histamine [108]. The membrane consisted of PVC as base polymer, an ionophore, an ionic additive, and a plasticizer. Ionophores promote the passage of ions through membranes upon capturing them in a reversible way. By creating host–guest complexes, ionophores such as cyclodextrin interact with the cationic biogenic amines and transport them into the membrane. Combined modifications can help to improve the characteristics of the electrode even further: Geto et al. modified a glassy carbon electrode with two modifiers, multi-walled carbon nanotubes (MWCNTs), and the conducting polymer p-AHNSA. Comparing the electrochemical signal between the bare and the modified electrodes, the authors found a 13-fold signal increase in the case of the MWCNTs modification and a 34-fold increase for p-AHNSA. Using both modifiers together as a hybrid layer, the signal was enhanced by a factor of 40 [99].

As a point of attention, one should be aware that nBRE-type sensors suffer from a lack of selectivity and nonspecific signals when it comes to BA detection in complex matrices. For example, Degefu et al. studied the influence of uric acid and histidine on the detection of histamine by square wave voltammetry using a lignin-modified glassy carbon electrode. In the presence of uric acid and histidine, the detection signal for histamine was reduced and the reduction correlated with the concentration of these interfering compounds [100]. Additionally, the biogenic amines tyramine and tryptamine are known to interfere with the electrochemical signal during the detection of histamine [105]. In such cases, prior to detection, sample preparation steps are needed to extract the target analyte.

Veseli et al. developed a histamine sensor based on cyclic voltammetry with working electrodes made of screen-printed carbon coatings modified with rhenium (IV)-oxide powders [109]. The ReO_2_ catalyst allows for histamine oxidation at overpotentials as low as −100 mV, which minimizes the risk for electrochemical conversion of matrix molecules. Using the peak current as output signal in a flow-injection setting, the LoD was 0.2 mg/L with a linear range up to 10 mg/L in 0.1 M PBS buffer [109]. The method rendered histamine concentrations (in the order of 900 mg/L) in a variety of fish sauces correctly as proven by spectrophotometric reference measurements. Still, the cross-selectivity to tyramine and tryptamine was substantial, while it was low for putrescine and cadaverine: This observation, together with the molecular structure of these BAs (see Figure 1), supports a proposed reaction mechanism between histamine and ReO_2_ in which the imidazole ring of histamine is involved, but not the amine group. The article by Veseli et al. provides also a literature overview on related electrochemical detection techniques and the respective electrode types. As a final example, Butwong and coauthors employed differential-pulse voltammetry for histamine detection using a glassy carbon electrode covered with multiwalled carbon nanotubes and Ag-AgO_2_ nanoparticles to catalyze the oxidation of histamine [110]. This sensor reached an LoD 0.18 μM, allowed to quantify histamine in fish sauces, and there was no measurable interference by putrescine and cadaverine. 

### 2.3. Towards BA Sensors with Bio-Recognition Elements

**Figure 4:**
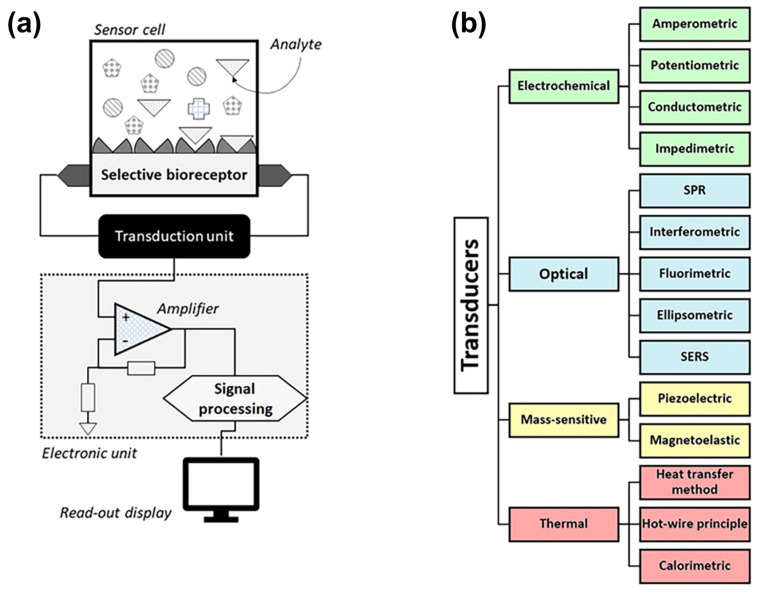
(**a**) Schematic representation of the key elements of a generic biosensor in which the analyte (target molecules) binds selectively to receptors, serving as biorecognition elements. The transducer converts the chemical recognition to a measurable signal that is amplified and processed to render information of the analyte concentration. (**b**) The established transducer principles for BA detection include electrochemical, optical, mass sensitive, and thermal sensors, each with a plurality of subtypes. Chemosensors without a biorecognition element belong mostly to the electrochemical category, and a few chemosensors are equipped with an optical detection scheme.

Biosensors, as a subclass of chemosensors, can be defined as analytical devices that convert a biological recognition response into a quantifiable signal (see Figure 4a for the basic design). The central idea is that the recognition should be selective at the molecular level to avoid nonselective response to similar or interfering compounds (see Section 2.2 above). Biosensors in the strict sense utilize recognition elements of biological origin, such as nucleic acids, proteins (including antibodies, enzymes lectins and peptide aptamers), organelles, bacteriophages [111], whole cells [112], and tissues [113]. Additionally, nucleic-acid based aptamers are considered as receptors of biological origin. Sensors that employ synthetic receptors such as molecularly imprinted polymers (MIPs) are addressed as biomimetic sensors [114]. Within this review, aptamer-based BA sensors are addressed in Section 3, MIP-based sensors are discussed in Section 4, and protein-based sensors (antibodies and enzymes) are the topic of Section 5. 

The physicochemical Interaction between the receptors and the molecular targets is converted into a measurable signal by the transducer, which is usually understood as the way of signal generation and not necessarily as a physical element. Although this signal is always electronic or optical in nature, there is a wide variety of transduction principles that differ in the way how the output signal of the sensor is generated. As illustrated in Figure 4b, there are four major categories of transduction principles used for BA detection, comprising electrochemical, optical, thermal, and gravimetric (mass sensitive) transducers. Each category contains a number of subtypes and combinations of transduction principles, such as electro-optical sensors, that exist as well. For a complete overview on the transducer-based classification of biosensors, we refer to the recent overview article by Naresh and Lee that discusses the signal-generation mechanism for each transducer type [115]. The output signal is usually amplified, converted from an analog to a digital format, processed, and displayed as a quantitative result0 (see Figure 4a). 

## 3. Biosensors for Biogenic Amines Using Aptamer-Type Receptors

### 3.1. Selection Techniques for Aptamers

**Figure 5:**
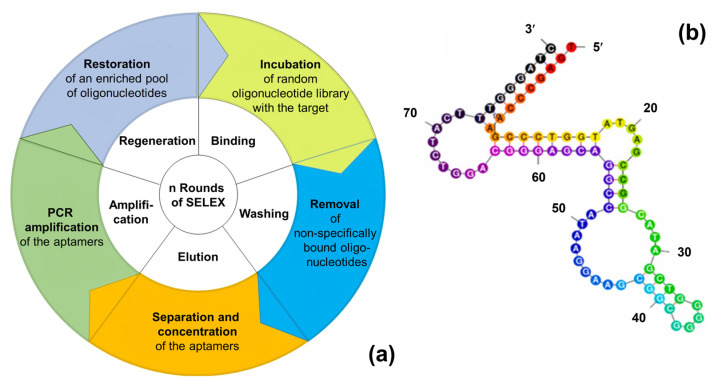
(**a**) Schematic representation of a SELEX process based on FluMag SELEX with the five process steps. (**b**) Illustration of the secondary structure with stems and loops of a tryptamine-binding aptamer developed in 2022. (**b**) is reprinted from ref. [122], Copyright 2022, with permission from Elsevier.

Discovered within two independent studies in 1990 [116,117], aptamers are single-stranded oligonucleotides or small oligopeptides, which can act as ligands for specific molecular targets. Aptamers can be selected in vitro with a process known as Systematic Evolution of Ligands by eXponential enrichment (SELEX), illustrated in Figure 5. During SELEX, aptamers are isolated from a combinatorial oligonucleotide library containing 10^12^–10^15^ random oligonucleotides. All these nucleotides consist of a variable, central region of 30–70 base pairs and two constant, flanking regions with a length of 20–25 base pairs. The incorporation of modifications in the random section of the oligonucleotides increases the structural diversity of the obtained aptamers. For example, inserting the non-natural nucleotide 7-(2-thienyl)imidazo[4,5-b]pyridine in the initial DNA library leads to aptamers with an up to 100-fold increased target affinity [118]. A typical SELEX for DNA aptamers consists of iterative cycles with the five steps illustrated in Figure 5a: (i) denaturation of the oligonucleotides, (ii) binding of the single-stranded DNA to the target, (iii) elution of the bound aptamers, (iv) PCR (polymerase chain reaction) amplification, and (v) retrieval of the DNA library. For the selection of RNA aptamers, two extra steps are required: After the elution of the target-bound RNA-aptamers and prior to PCR amplification, a reverse transcription is carried out. The amplification is followed by an in vitro transcription step to retrieve the pool of RNA oligonucleotides. Usually, 8–12 repeats of the above process and negative selection steps are necessary to obtain aptamers with high affinity for the target molecules.

The figure of merit of an aptamer for a given target is the dissociation constant *K_D_* (the inverse of the affinity constant, *K_A_*^−1^), which ranges from micromolar (mM) to nanomolar (nM), the latter being similar to the affinity of antibodies. The number of cycles can be extended up to 15 cycles or limited down to a single cycle. Accordingly, the SELEX process can last from a few weeks up to several months. Efforts have been made to automate SELEX by using a robotic set-up that can carry out parallel aptamer selections, thus minimizing the time [119]. The conventional SELEX process has been modified in numerous ways throughout the last two decades. New methods have been incorporated in the standard process such as high-throughput sequencing technologies and techniques for the easy separation of aptamer–target complexes from unbound oligonucleotides by, e.g., capillary electrophoresis, chromatography, or magnetic separation methods such as FluMag SELEX [120,121].

Nucleotide aptamers capture their targets by adopting a three-dimensional conformation: Aptamers fold initially into small helices and single-stranded loops (see, e.g., Figure 5b for the secondary structure of a recently developed tryptamine-binding aptamer [122]). Upon binding to the targets, they are further folded into tertiary structures. Aptamers can then form stable complexes with the target through hydrogen bonding, van der Waals forces, and electrostatic interactions [123]. Due to the variability of the secondary and tertiary structures, aptamers can recognize a large variety of structurally different molecular and even supramolecular targets. For an overview on their bioanalytical applications, including diagnostic and prospective therapeutic applications, we refer to refs. [124,125,126].

### 3.2. Aptamers for Selective Biogenic Amines Recognition

With respect to biogenic amines, a limited number of aptamers has been reported, namely for histamine, dopamine, tyramine, tryptamine, spermine, and ethanol-amine. Table 1 provides an overview that includes the selection method, the underlying nucleic-acid type, and the equilibrium dissociation constant *K_D_*; for the exact sequence of these aptamers, we refer to the original publications. Valenzano et al. combined SELEX with next-generation sequencing for the selection of DNA aptamers recognizing tyramine [127]. The authors applied a stringent SELEX protocol with multiple counter- and negative selection steps to select high-affinity aptamers. FluMag-SELEX has been employed to isolate DNA aptamers that bind to ethanolamine [128]. However, these aptamers were not specific for ethanolamine since they also had a strong affinity to structurally related amines, such as di- and tri-ethanolamine, methylamino-ethanol, and phenoxylethylamine [129]. Oguro and coworkers used resin-based SELEX for the selection of two different RNA aptamers binding to spermine [130]. The aptamers were evaluated for their affinity towards spermine and similar linear polyamines, such as spermidine and putrescine, using a resin-binding assay. The binding of the aptamers to spermine-analogous BAs was found to depend strongly on the structural length and the number of the primary- and secondary amine groups of the compound. These aptamers displayed a preference for BAs with a similar number of carbon atoms and amine groups as spermine.

Mannironi et al. used dopamine-immobilized agarose columns to select RNA sequences for the recognition of dopamine, a physiologically important neurotransmitter [134]. Among them, one RNA aptamer was evaluated for its specificity to dopamine by batch-affinity chromatography. This aptamer displayed also affinity to structural analogues of dopamine, being norepinephrine and L-DOPA but with less than half of the affinity to dopamine. For other catecholamines under test, the dopamine aptamer demonstrated low affinity. Later, an attempt was made to improve the dopamine aptamer by replacing the RNA sequence with its DNA homolog [135]. DNA aptamers are more robust against thermal and enzymatic degradation and less expensive to synthesize than their RNA counterparts. By monitoring the binding affinity of the DNA aptamer with fluorescence anisotropy, the equilibrium dissociation constant was found to be half of the RNA aptamer, hence a step in the right direction. The DNA homolog was also found to exhibit a slightly higher affinity for norepinephrine than dopamine. In a study from 2017, the performance of the DNA-based aptamer in comparison to its RNA version was re-evaluated: Using cyclic voltammetry with electrodes functionalized with the respective DNA- and RNA aptamers, it was clarified that the DNA-based aptamer is unspecific for dopamine while the RNA aptamer is indeed selective [136]. When tested against the other catecholamines norepinephrine, L-dopa, and catechol, the DNA aptamer could not discriminate between these molecules.

Keeping these elements in mind, it becomes clear that still research is required to develop aptamers for biogenic amines in general and that selectivity is an issue that requires ongoing attention. For biosensing applications, it is good to know that aptamers are resilient against elevated temperatures up to 95 °C and strong pH- and salinity variations of the sample under study. Nevertheless, their target selectivity relies on their exact conformation and optimal performance requires therefore well-defined temperature, pH, and buffer composition settings. These factors depend directly on the conditions under which the aptamer was selected; in turn, the selection process can be adapted in advance to the envisaged bioanalytical application. A complication that shows up when analysing food matrices, cell lysates, or blood serum is the enzymatic degradation of aptamers by nucleases: To reduce the nuclease sensitivity, it is possible to employ chemical modifications, such as a phosphonothioate backbone and 2′-O-methyl substitution [137,138]. Additional nucleic-acid analogs with enhanced stability against enzymatic degradation are reviewed in ref. [139]. Knowing about these pitfalls and ways out, aptamers can be valid alternatives to antibodies (see Section 5), with the benefit that aptamers can be selected for non-immunogenic targets, which is still impossible to achieve with their antibody counterparts. Furthermore, aptamers lend themselves for labeling, which will be discussed in the next section.

### 3.3. Aptasensors for BA Detection with Optical Transducers

**Figure 6:**
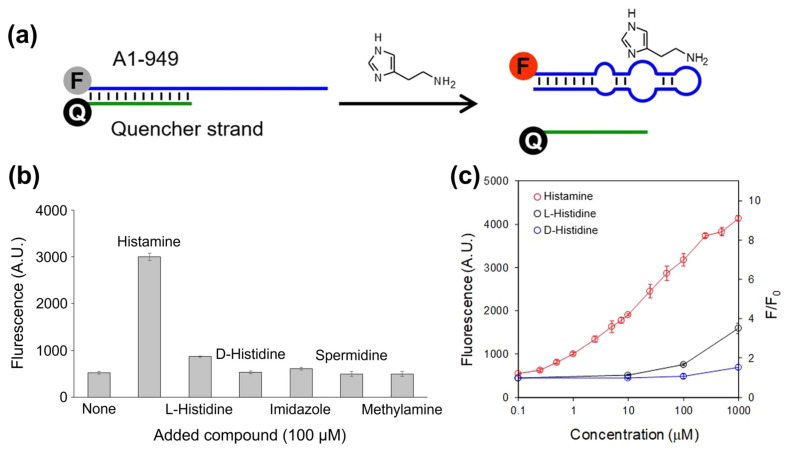
(**a**) Fluorescence-based assay for the detection of histamine in solution. The aptamer carries a Cy5 fluorophore at the 5′ terminus and is hybridized with a partially complementary quencher strand with a BHQ2 molecule at its 3′ location [142]. Recognition of histamine removes the quencher strand and the aptamer attains a new conformation, resulting in fluorescence emission. (**b**) The selectivity test reveals high affinity of the aptamer to histamine and reduced affinity to histidine and other biogenic amines, here for high concentrations of 100 μM. (**c**) Up to target concentrations of 10 μM, the response to the two histidine enantiomers is negligibly small. Figure adapted from ref. [142], Creative Commons CC BY license.

All biosensors utilizing aptamers as recognition elements are commonly addressed as “aptasensors”. In the context of food safety analysis, aptamers are most often developed for the detection of residues of pesticides and antibiotics (see, e.g., references [140,141]). During recent years, aptamers came also in the focus for biogenic amines and there are several examples of aptasensors employing optical transducers: Figure 6a shows the basic concept with an aptamer (RNA, 37 bases) that is functionalized with a Cy5 fluorophore at the 5′ position [142]. Due to hybridization with a partly complementary quencher strand (the quencher molecule at the 3′ position), the fluorescence signal is suppressed. Recognition of histamine molecules in the solution results in strand displacement and high fluorescence signals of Cy5. Interestingly, the cross-selectivity to competitors is low and exists only at high target concentrations (Figure 6b). The sensor response is widely linear with respect to the *log* of the histamine concentration with a detection limit of 1 μM. The principle was furthermore validated with spiked and intentionally aged tuna samples, and the justness of the sensor-derived concentrations was confirmed by reference measurements with a histamine-dehydrogenase kit. Additional information can be found in Table 2, summarizing key data on a variety of selected BA aptasensors with optical or electrochemical readout.

Another example at the optical side are histamine sensors using a colorimetric principle based on light scattering [143]: Solutions of gold nanoparticles (AuNPs) have a reddish colour and, after addition of salts, the AuNPs form aggregates, which results in a blueish colour. When aptamers are present in the solution, they bind loosely to the AuNP surfaces, which suppresses particle aggregation. In turn, when adding histamine, the aptamers bind preferentially to the histamine, the AuNPs are unshielded, and therefore discoloration to blue takes place. These effects can be measured quantitatively in a concentration-dependent way by UV–Vis absorption spectroscopy and Mairal Lerga et al. obtained detection limits of 8 nM in tuna- and sardine extracts. The same strategy was employed for detecting spermine in urine samples [144]. To best of our knowledge, there are no optical aptasensors documented in the literature for the BAs tyramine, tryptamine, cadaverine, and putrescine.

When looking at BAs beyond the food safety context, numerous aptasensors have been developed that are targeting dopamine due to its clinical relevance as a neurotransmitter and hormone. Dopamine is derived from the amino acid tyrosine as its precursor. The molecular structure of dopamine resembles the heterocyclic and aromatic BAs of Figure 1, meaning that aptasensing strategies for dopamine are transferable to the food-related biogenic amines. Zheng et al. developed a colorimetric assay based on light scattering at AuNPs with a LoD of 0.36 μM dopamine in a 40 mM NaCl solution [145]. The blue shift was measured from UV–Vis absorption at two different wavelengths (524 and 660 nm). The sensor response to competitors and metabolites of dopamine was 2–5 times lower than the specific response. As a limitation, the salinity of the sample requires a stringent control. In a variation to this method, Xu and coworkers combined the gold-nanoparticles aggregation with the FRET (Förster resonance energy transfer) principle [146]. Gold nanoparticles, protected against aggregation by an aptamer layer, quench the fluorescence emission of Rhodamine B. When the aptamers bind instead specifically to dopamine, the AuNPs aggregate and the FRET mechanism between Rhodamine B and the NPs is suppressed; hence, the fluorescence signal of Rhodamine B recovers. The authors reported a calculated LoD of 2 nM and were able to detect spiked dopamine in the order of 10 mg/kg in extracts of swine feed and chicken liver. In another example for optical dopamine assays, CdTe quantum dots (QDs) acted as fluorophores and poly–pyridine ruthenium complexes (Ru(bpy)_2_dppz]^2+^) served as fluorescence acceptors [147]. In the absence of dopamine, the aptamers ligate to the Ru complexes and the fluorescence signal of the quantum dots is freely emitted. When dopamine is present, the aptamers bind to the dopamine molecules and the negatively charged QDs assemble at the Ru complexes, which quenches the fluorescence signal. A detection limit of 19 nM was reached in PBS buffer, and the assay remained feasible in 100× diluted fetal bovine serum.

**Table 2:** Overview of selected biogenic-amine sensors utilizing aptamers as receptors, together with the sensor architecture and the underlying transducer principle. In the case of food items, the samples are aqueous extracts. The limits of detection (LoDs) are used as specified in the original publications, which holds also for the analytically useful range.

Biogenic Amines	Sample	Sensor Architecture	Transducer	LoD	Analytical Range	Reference
Histamine	Tuna samples	Fluorescently labeled RNA apta-mer, quencher displacement	Fluorescence of Cyanine 5	1 µM	1–1000 µM	[142]
	Tuna and sardines	Aptamer adsorbed at gold NPs, salt-induced aggregation	Colorimetric (UV–Vis)	8 nM	8–2000 nM	[143]
	Buffer, synth. urine	Competitive assay with histamine and histamine-coated magn. beads	Colorimetric (UV–Vis)	18 pM in buffer,	N/A	[132]
	Spiked PBS solution	Biotinylated aptamers are attached to streptavidin-modified surfaces	Impedimetric (faradaic)	4.83 mM	4.83–60 mM	[131]
Spermine	Urine	Aptamers adsorbed on AuNP, aggregation in presence of spermine	UV–Vis absorption	473 nM	0–18 µM	[144]
Dopamine	Swine feed, chicken liver	AuNPs solution in combination with the fluorophore Rhodamine B	FRET-based fluorescence	2 nM	26 nM–2.9 μM	[146]
	Human body fluids	Thiolated aptamer and ferrocene immobilized on gold electrode	Cyclic voltammetry, Impedance	60 pM, 20 pM	0.1–10 nM	[148]
	Human serum	Amplification based on Ce-MOF and methylene blue	Square wave voltammetry	6 pM	6 pM–100 nM	[149]
	Filtered serum	Amplification by AgNP solution with nanocarbon modification	Diff. pulse voltammetry	700 pM	3–110 nM	[150]
	Human blood	Thiolated aptamer immobilized on gold electrode	Square wave voltammetry	1 nM	5–150 nM	[151]
Ethanol-amine	Tap water, serum	G-quadruplex films immobilized on gold electrode	Impedimetric (faradaic)	0.08 nM	0.16–16 nM	[152]

### 3.4. Aptamer-Based BA Sensors Based on Electrochemical Transducers

**Figure 7:**
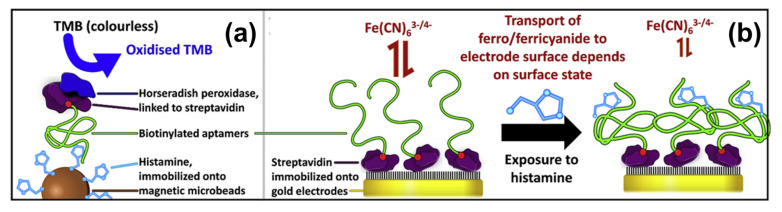
(**a**) Functionality testing of a histamine aptamer; histamine is tethered to magnetic beads while the biotinylated aptamer is linked to horseradish peroxidase HRP [131]. The aptamer-histamine binding is visualized in a colorimetric way through the enzymatic oxidation of TMB (tetramethylbenzidine) molecules by HRP. (**b**) Electrode design for impedimetric histamine detection with the aptamer immobilized on gold electrodes. Before binding of histamine, the electrode is accessible for the redox couple Fe(CN)_6_^3−/4−^, corresponding to a low impedance value. Upon recognition of histamine, the tertiary structure of histamine changes, resulting in a blocking of the electrode, which increases the charge-transfer resistance and the impedance value. Figure reprinted from ref. [131], Copyright 2020, with permission from Elsevier.

In contrast to the optical methods described above, electrochemical sensors require that the aptamer is immobilized on a solid electrode; gold electrodes and thiol-modified aptamers are a popular combination due to the quasi-covalent Au–sulfur bonds. Spacer groups can be utilized between the electrode and the actual aptamer to minimize steric hindrance during bio-recognition of the target by the receptor. In a first example, Ho et al. developed a DNA aptamer with 49 nucleotides in the variable region that was validated in an optical assay shown in Figure 7a [131]. Due to its similarity with the ELISA concept, this approach was termed ELONA (enzyme-linked oligonucleotide assay), resulting in LoD values of ca. 100 nM in spiked PBS buffer. For impedance spectroscopy (see Figure 7b), the aptamer is immobilized on a gold electrode, and the sensing effect is based on blocking the accessibility of the electrode for the redox mediator ferro/ferricyanide Fe(CN)_6_^3−/4−^, hence an increase of the impedance signal. This is an example of faradaic impedance spectroscopy; the non-faradaic variant is addressed in Section 4.3 on MIP-type receptors. For buffer solutions, ref. [131] mentions an LoD of 4.83 mM (see Table 2) and negligible cross-selectivity to histidine. In a similar faradaic concept based on Fe(CN)_6_^3−/4−^, Liang et al. detected ethanolamine down to concentrations below 1 nM in spiked water and serum samples [152]. The aptamer was rich in guanine bases to form a G-quadruplex structure in presence of K^+^ ions, and, due to the conformational change upon target binding, the charge-transfer resistance increased as a function of the ethanolamine concentration.

A variety of voltametric methods has been developed for the electrochemical detection of dopamine (see Table 2). Bahrami and coworkers employed differential pulse voltammetry with an LoD of 700 pM dopamine in buffer and spiked concentrations from 10–60 nM were rendered correctly in protein-free human serum [150]. To reach such low detection limits, they functionalized glassy carbon electrodes with hybrid nanoparticles made of silver, carbon nanotubes, and graphene oxide. The aptamers (58 bases) were linked covalently to the hybrid particles. The sensing effect relies on the oxidation of H_2_O_2_, that is catalyzed in presence of silver. Upon the binding of dopamine to the aptamers and the resulting change in their tertiary structure, H_2_O_2_ is shielded against the Ag particles and the oxidation current decreases. In a concept without redox-active chemicals, carbon electrodes were functionalized with gold nano-stars, offering a large surface for aptamer functionalization. Binding of dopamine to the aptamers shielded the Au nano-stars, resulting in a concentration-dependent increase of the charge-transfer resistance *R_ct_* [153].

Azadbakht et al. developed a cyclic voltammetry method for dopamine detection, which resembles the strand displacement concept shown in Figure 6a [154]. The aptamer (58 nucleotides) was hybridized with short capture-DNA fragments (12 bases), that were immobilized on a glassy carbon electrode modified with AuPt nanoparticles and carbon nanotubes. Methylene blue (MB), serving as redox mediator, assembled with the DNA duplexes thanks to the MB–guanine interaction [155]. Upon adding dopamine to the supernatant, the aptamers left the electrode surface together with the MB molecules. The resulting drop in peak current allowed to detect dopamine quantitatively and in a linear way for concentrations from 1 to 30 nM in 0.1 M PBS buffer with a detection limit of 0.22 nM [154]. Zhang et al. fabricated a dopamine aptasensor using an amplification strategy based on electrodes modified with highly conductive Ce-MOF particles (cerium metal-organic frameworks) and m-PdNFs-G4-MBs nanostructures as signal enhancer. The specific dopamine aptamers were tethered to the electrodes via hybridization with small ssDNA fragments bound on the Ce-MOF. In presence of dopamine, the aptamer binds to the target and detaches from the ssDNA. Then, the ssDNA captures the signal enhancer nanostructure decorated with the complementary ssDNA. Using cyclic voltammetry, they reported a limit of detection down to 6 pM for dopamine [149]. Interestingly, voltametric sensors do not necessarily require noble metal electrodes or nanoparticles: As shown by Wang et al., also conjugated polymers such as PEDOT fit the purpose, enabling reportedly sub-picomolar detection limits [156].

Regarding the amperometric detection of dopamine, Liu and coworkers utilized an electrochemical cell in which the working electrode (glassy carbon) was modified with gold nanoparticles [157]. The aptamer was coupled to the Au nanoparticles via thiol coupling and an additional blocking layer served to suppress nonspecific sensor response in complex samples. After binding of dopamine to the aptamer, the dopamine was oxidized electrochemically to dopamine quinone, and instantly reduced back to dopamine in a chemical way using TCEP (tris(2-carboxyethyl)phosphine). This circular reaction results in an amplification of the anodic current with an estimated LoD of 1.8 nM in PBS buffer and correct concentration data in dopamine-spiked human blood serum that was diluted with PBS. The assay was performed in two subsequent steps with first the dopamine binding from the serum samples, followed by the actual amperometric measurement in pure PBS, enriched with TCEP molecules.

## 4. Biomimetic Sensors for Biogenic Amines Using Molecularly Imprinted Polymers

### 4.1. Molecular-Imprinting Technology

**Figure 8:**
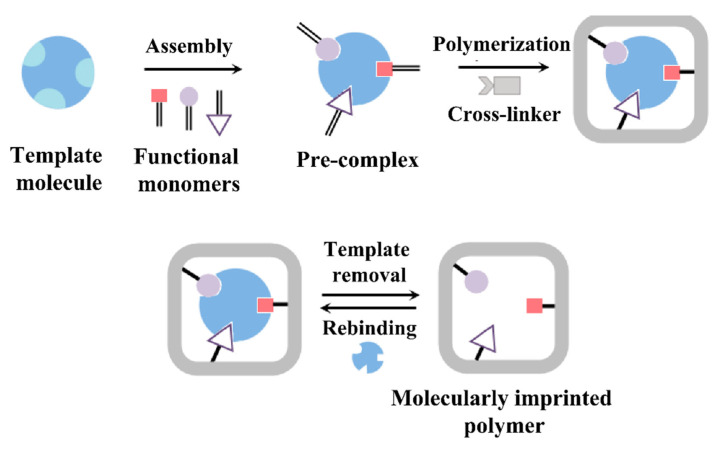
MIP receptors rely on the principle of host–guest chemistry. Functional monomers are polymerized in the presence of template molecules with which they form weak, sterically oriented bonds. After template extraction by organic solvents, the MIP cavities bind target molecules from aqueous media in a reversible fashion. Figure reprinted from ref. [175], open access article distributed under the terms and conditions of the Creative Commons Attribution (CC BY) license.

Molecularly imprinted polymers (MIPs) are synthetic polymers that can be used for the recognition of biological and other organic compounds; therefore, they are also addressed in the literature as plastic antibodies [158,159]. MIPs act as artificial receptors that recognize a wide range of targets, including amino acids and their derivatives [160,161,162], proteins and peptides [163,164,165], and environmental pollutants such as pesticides and antibiotics [166,167,168]. Regarding surface-imprinted polymers (SIPs) that are often utilized for the detection of larger biological targets such as viruses, cells, and bacteria, we refer to a review article by Eersels and coworkers [169]. From the manufacturing point of view, synthesis of MIPs is convenient, given the fact that they can be produced with a variety of established polymerization protocols [170,171]. Molecular imprinting techniques are based on the formation of a three-dimensional polymer matrix which contains micro- or nano-cavities that are specific to the target molecules that correspond in most cases, to the so-called template molecules utilized during the imprinting step. The polymers are created by copolymerization of functional monomers in the presence of the template and a cross-linker that stabilizes the created polymer network (see Figure 8 for a schematic illustration). Commonly, organic pyrogens are added to this polymerization mixture to allow for a facile extraction of templates after polymerization and an easy accessibility of the receptor pockets for target molecules. During the actual polymerization, the monomers self-assemble around the templates under the influence of weak electrostatic interactions such as hydrogen bonds and van der Waals forces. Thus, the preferred environment for polymerization is hydrophobic and in the early molecular-imprinting protocols, polymerization was indeed performed in organic solvents. It is a challenging fact that many biological molecules are soluble in aqueous media while they can deteriorate in organic solvents, which holds especially for sensitive targets such as peptides and proteins [172]. To overcome this limitation, also hydrogels made their way to molecular-imprinting technology [173,174].

After the formation of the polymer matrix, the templates are extracted, leaving behind micro-cavities inside the polymer, which are complementary to the targets in terms of size and chemical groups. The micro-cavities act as recognition sites that will bind the targets in a reversible fashion. The physical characterization of MIPs usually addresses their multiscale morphology, porosity, and swelling degree; their functional groups (by spectroscopic techniques); and their thermal stability [176]. To evaluate the performance of MIPs as synthetic receptors, the key parameters are their binding capacity, affinity, and selectivity [177,178]. Widely employed for this purpose are batch-rebinding experiments using optical UV–Vis absorption spectroscopy. The binding isotherms usually display a Langmuir- or Freundlich-type behavior, the latter reflecting a heterogeneity in the target-binding affinity of the receptor sites (see, e.g., [179,180]). A recently developed MIP material with high affinity for glucose has a *K_D_* value of 3·× 10^−8^ M [181]; this comes close to antibodies and is in a similar range as reported for BA-sensitive aptamers in Table 2. Specifically for histamine, Feng et al. developed a MIP with *K_D_* as low as 0.89 nM, illustrating that MIPs and aptamers are not necessarily inferior to more classical receptors [182]. In addition, MIPs offer a variety of advantages, including (i) facile long-term storage at ambient conditions without deterioration of their affinity; (ii) repetitive usage after extraction of captured molecules; (iii) resilience to many organic solvents; and (iv) operation under non-physiological conditions regarding temperature, pH, and ionic strength of the liquid under study.

### 4.2. MIP-Type Receptors for Biogenic Amines

Regarding biogenic amines, MIPs have been successfully synthesized for the binding of histamine [183], tyramine [184], tryptamine [185], putrescine [186,187], and dopamine [188]. Especially, histamine MIPs are well studied: They exhibit not only a higher affinity for histamine than for structurally related compounds (histidine, 5-hydroxy tryptamine, 5-hydroxyindole-3-acetic acid, and 1H-imidazole-1-acetic acid, see ref. [189]), but also with respect to the anti-histaminic drugs ranitidine and dimentidene [190,191], the neurotransmitters serotonin and dopamine [192], and other BAs [191]. For cross-selectivity testing against L-histidine, a variety of transducers has been employed including impedance spectroscopy [183,193], the heat transfer method HTM [194], and surface-plasmon resonance SPR [189]. A negligibly low cross-selectivity with respect to the histamine-precursor L-histidine is especially important in the food-analysis context given the fact that most foodstuff spoiled with histamine contains naturally high amounts this amino acid. Tryptamine-imprinted polymers show a seven-fold stronger response to tryptamine than to tryptophan [195], which is structurally identical to tryptamine, except for the additional carboxylic group that tryptophan contains in its side chain. Similarly, tyramine-MIPs are more selective for tyramine than for dopamine, L-dopa, and L-tyrosine, which differ from tyramine in only one or two functional groups [184].

As mentioned above, MIPs are advantageous over other bio-recognition elements (such as aptamers, but especially enzymes and antibodies) when it comes to chemical degradation. Although they retain their structural integrity in a wide range of pH values, their target-binding affinity is pH-dependent due to the different protonation states in which not only the target molecules but also the monomers of the MIP material can occur. Histamine can form up to two hydrogen bonds with the surrounding MIP cavity, and, depending on the pH of the solution, histamine molecules are mainly neutral (HisN, pH above 10.4), double-protonated (His++, pH below 6.9), and single-protonated His+ for pH values in between [196]. Consequently, the pH of the sample affects the binding probability due to the fact that hydrogen bonds only form when a protonated position at the target molecule links to a deprotonated site inside the MIP cavity or vice versa (compare also Figure 8). Changes in the pH of the solution affect the ability of MIPs to bind Bas, since they interact by noncovalent bonding: The interaction of MIPs with histamine is based on hydrogen bonding for aqueous solutions and on electrostatic forces in the case of organic solvents [191,193]. Tests with histamine MIPs in an aqueous environment indicate that maximum detection signals and, hence, sensitivity occur at a pH close to the first p*Ka* value (acid dissociation constant) of histamine (pH 6.9), when histamine is mostly in its single-protonated form [189,191,193].

**Figure 9:**
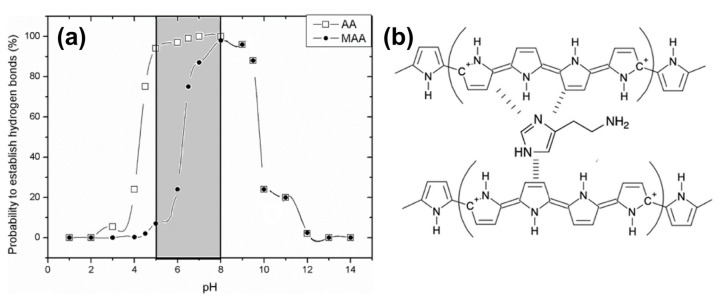
(**a**) Combinatorically calculated pH dependence of the binding probability between histamine and MIPs synthesized from acrylic acid (AA, open boxes) and methacrylic acid (MAA, solid dots) based on the variety of protonation states of the target molecule and the MIP receptors. Reprinted with permission from ref. [196]. Copyright 2013 American Chemical Society. (**b**) Structure of the conducting polymer polypyrrole (PPy) with a histamine molecule trapped by π–π stacking, this interaction is reversible and independent of the pH value of the sample. Reprinted from ref. [197]. Copyright 2021 American Chemical Society.

For histamine testing in a medical context (e.g., analysis of intestinal liquids) but also with respect to food sample extracts is useful to widen the analytically accessible pH range. Peeters et al. developed an experimentally confirmed combinatorial model in which also the p*Ka* values of the MIP monomers were taken into account [196]. This way, one can analyze for each pH the probability of having zero (non-binding), or one, or two hydrogen bonds (binding situations) between histamine molecules and the MIP cavities. MIPs made of methacrylic acid monomers (MAA has p*Ka* 6.5) by bulk polymerization bind histamine efficiently between pH 6 and pH 10, as shown in Figure 9a. With MIPs synthesized from acrylic acid with its lower p*Ka* of 4.5, the useful pH range extends down to pH 4. This illustrates that the suitable choice of the monomer is crucial when MIPs are developed for analyzing samples with a non-neutral pH. In a recent work, histamine MIPs were developed based on electro-polymerization of pyrrole to polypyrrole, a conjugated, electrically conducting polymer shown in Figure 9b. The host–guest interaction relies primarily on π–π stacking, which is widely insensitive to pH, except for strong acids and bases [197]. Electro-polymerization can be applied to numerous monomers and blends thereof [198], allowing for a comparatively facile synthesis directly at the surface of metallic electrodes, which will be discussed in the following subsection. The laser grafting of MIP layers by atom transfer radical polymerization offers the same benefits and may become popular in future for the pointwise functionalization of microelectrode arrays with MIP layers [199].

### 4.3. MIP-Based BA Sensors with Mass-Sensitive Transducers

The majority of publications on mass-sensitive transducers refers the quartz-crystal microbalance QCM and ref. [200] by Diltemiz et al. reviews the combinations of MIP-type receptors with the QCM technique in a broad perspective. Regarding BA detection, Pietrzyk et al. synthesized histamine MIPs directly by electro-polymerization on the platinum electrodes on the QCM chips using derivatives of bis(bithiophene) monomers [201]. The crucial factor was a double-layer structure of the polymer, consisting of a nonimprinted bottom layer and a histamine-imprinted capping layer: This concept avoids direct contact between the template molecules and the platinum surface, which would result in an over-oxidation of the templates during electro-polymerization. After template extraction with NaOH, the authors found a sensitivity corresponding to a drop of the resonance frequency by 0.33 Hz per mM histamine (for a studied range from 10 to 100 mM) under flow injection conditions with HEPES buffer as carrier medium. The calculated detection limit under optimized conditions was 5 nM, and the affinity for histamine was at least five times higher than for the related compounds dopamine, tryptamine, and imidazole. Horemans et al. developed a QCM-based assay in which MIP micro-powders (25 μm mesh) were deposited on the QCM electrodes by soft lithography and a thermal treatment using thin PVC (polyvinyl chloride) layers as an adhesive [183]. The lowest detected concentration was 1 μM of histamine in water with an average sensitivity of 0.27 Hz per μM in the range up to 100 μM. This sensitivity is almost a thousand times higher than for the electropolymerized MIP layer of ref. [201], although the micro-powders reached only ≈30% surface coverage on the QCM electrode. Horemans et al. optimized the polymerization mixture with respect to the molar ratios of template molecules, methacrylic acid monomers, and EGDM (ethylene glycol dimethacrylate) as a cross-linker. For the optimal composition, the MIP material displayed 524 μmol of available histamine-binding sites per gram of MIP powder, as determined by batch-rebinding experiments with a Freundlich analysis of the corresponding isotherms [183,202]. Table 3 summarizes the characteristics of all MIP-based BA sensors addressed within Section 4, allowing also for a comparison between the transducer principles.

**Figure 10:**
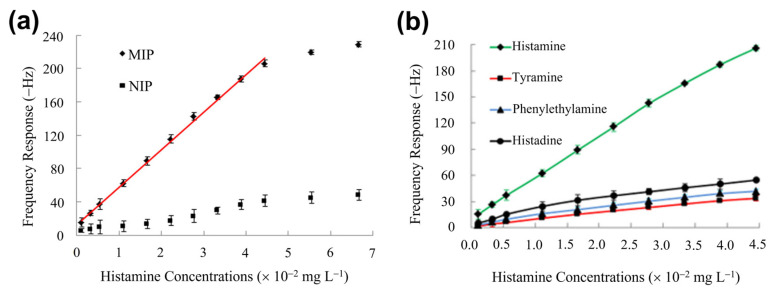
(**a**) Dose–response curve of histamine detection obtained with a quartz-crystal microbalance with a MIP-functionalized QCM chip. The response is linear and shows saturation for higher concentrations [203]. Coatings made of a nonimprinted reference polymer (NIP) cause a weak, nonselective response due to adsorption without molecular recognition. (**b**) QCM crystals functionalized with the histamine–MIP show a small response to competitor molecules in the same order as the NIP signal in panel (**a**). Figures adapted with permission from ref. [203], Copyright 2014, American Chemical Society.

Dai et al. developed a QCM sensor in which the histamine MIP was prepared by sol–gel synthesis from a blend of silanes, resulting in optimal binding capacity at pH 7.6 [203]. The MIP material was dissolved in dichloromethane containing PVC as a binder, drop-casted on QCM chips, and finally dried. Figure 10a shows a substantial frequency response of the QCM device with respect to histamine concentrations in PBS at concentrations that are subcritical for human health. Using a nonimprinted polymer (NIP) coating on the crystals gave a weak, nonspecific response. While this illustrates the selectivity of molecular recognition, one may assume that the response of the MIP coating is to a small part also caused by nonselective adsorption of target molecules. In very recent publications (see Section 4.4 and, e.g., [197]), authors have employed a differential signal between the active channel (MIP) and the passive channel (NIP) to determine the signal change truly through molecular recognition. As shown in Figure 10b, the cross-selectivity to competitor molecules is four to five times lower as compared to selective recognition [203]. Then, canned fish samples, including mackerel, sardines, salmon, saury, and tuna were homogenized; proteins and fats were extracted in a series of preparation steps; and the dried fish extract was dissolved in PBS. The histamine concentrations found in these samples (in the order of 0.1 to 0.7 mg/kg) agreed well with a HPLC reference analysis. Moreover, in fish extract samples with spiked histamine, the total concentrations were rendered correctly with deviations of a few percent at most. In this and in related publications, the ratio between the detected and the real concentration is quantified by the recovery factor, for which Dai et al. reached 93.2–100.4%, underpinning the accuracy of this QCM sensor platform.

### 4.4. MIP-Based BA Sensors with Electrochemical Transducers

The first work on MIP-based, impedimetric histamine detection was published already in 2010 by Bongaers et al. [193]. They immobilized MIP micro-powders based on methacrylic acid on coplanar gold electrodes using the polymer OC_1_C_10_–PPV (poly-phenylene vinylene) as a conductive adhesive, enabling the use of non-faradaic impedance spectroscopy. In spiked PBS buffer, the LoD was 2 nM of histamine and a concentration of 12 nM was sufficient to increase the impedance signal by 50%. Interestingly, impedimetrically determined LoD values did not much decrease in more recent publications, but vice versa it is also not essential to detect sub-nanomolar BA concentrations in food safety analysis. Using the technique of ref. [193], Horemans et al. reported an LoD of 1 nM in buffer and a zero response towards the competitor histidine; furthermore, they demonstrated that NIP coated electrodes show no response to histamine [183]. Using differential sensing between MIP- and NIP-coated electrode couples to compensate for electrode fouling, the authors analyzed filtered liquid from canned tuna. The native concentration was 4 ppm and thus below the critical level of 50 mg/kg, as specified in the US food regulations. The fluid was spiked with histamine concentrations up to 250 ppm and the spiked fluid was diluted with a 1000-fold higher volume of PBS buffer, thus resulting in concentrations from 4 to 250 parts per billion. Even the lowest concentration gave a signal above uncertainty, meaning that the combination of MIPs with impedance analysis is well suited to quantify histamine in fish extracts in a fast and sensitive way. To put these concentrations in perspective, 1 ppm of histamine in an aqueous sample corresponds to 55 μM and 1 ppm to 55 nM. To our knowledge, there is no reported work yet on impedimetric histamine detection directly in fish filets or untreated fish extracts. Other BA sensors utilizing MIPs in combination with electrochemical readout in a broad sense are included in Table 3.

**Figure 11:**
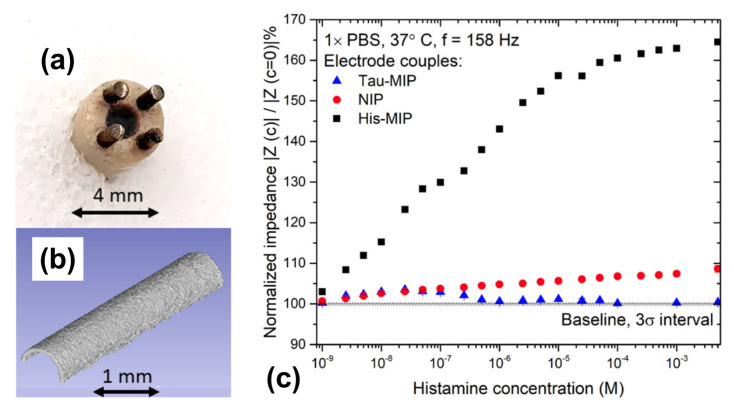
(**a**) Four-electrode configuration with two titanium pins coated with histamine-imprinted Ppy and two pins with a NIP coating for differential sensing in complex samples. (**b**) Optical coherence tomography reveals homogeneous Ppy coating with 1 μm thickness. (**c**) The dose–response curve reveals an especially wide analytical range of the MIP electrodes to histamine and a negligible signal increase when the same histamine concentrations are applied to the NIP electrodes. For comparison, MIP layers were also synthesized with a taurine-sensitive coating, which did not show a quantifiable response to histamine, even for the highest concentrations. The figure is reproduced with permission from ref. [197], Copyright 2021 American Chemical Society.

As a relevant point, there is a growing interest of the medical field in measuring the histamine concentration within the human intestines (mainly in the duodenum) as an indicator for chronical inflammations and the irritable bowel syndrome IBS [215]. Based on work by Ratautaite et al. in ref. [204], Wackers and coworkers designed a sensing probe that is small enough to fit into a nasogastric catheter, as shown in Figure 11a [197]. The electrodes consist of titanium pins that are coated with electropolymerized polypyrrole; two pins were coated with histamine-imprinted PPy, and the two other pins had a NIP layer to allow for differential sensing in complex fluids. This design is symmetrical in the sense that there is no distinction between working- and counter electrode and the probe design allows to simply dip the electrodes into any sample with large enough volume. Electro-polymerization offers the possibility to functionalize a large number of electrodes simultaneously under identical conditions, which makes this route suitable for upscaling to batch fabrication. The coating layers have a homogeneous morphology, as seen in Figure 11b. In PBS buffer, the sensor has an analytical range from 1 nM up to ca. 2 mM (see Figure 11c**)** and, in highly complex intestinal liquid concentrations, could be quantified from 25 nM up to 1 μM. Another sensor with a dip-in design for food analysis was published recently by Stilman and coworkers for detecting yeasts and proteins in yoghurt and malt extracts; an application on histamine detection in fish samples is expected shortly [216].

As a variation to impedimetric sensing, Croux et al. developed a concept towards wireless histamine detection by admittance spectroscopy [205]: The idea is to monitor the histamine contents of food samples from a distance, especially through packaging foils, by packing the food product together with passive radio frequency identification (RFID) tags. A planar LC-resonator circuit was prepared from conductive silver inks on PET (poly(ethylene terephthalate)) foils, and the sealed tag had an opening on the capacitive element onto which MIP powder was attached with PVC adhesive. The binding of histamine by the MIPs changes the dielectric properties of this coating and the resonance frequency of the LC circuit alters in a concentration-dependent way. By probing the resonance shift with an antenna, the authors obtained an analytical range from 50 to 800 nM histamine in PBS over a distance of several centimeters and through a glass plate between the RFID tag and the detection antenna. There was no follow-up work on admission spectroscopy, but wireless biosensor patches are an emerging trend with considerable perspectives [217]. Besides wireless transmission, color reactions are also potential candidates to identify BA spoilage of packaged food products such as meat. Technical concepts of chromogenic sensing were proposed by Steiner et al., and the current state-of-the-art practices was summarized in a review by Miller and coworkers [218,219]. For packaged meat products, Alexi et al. reported a slightly more invasive concept: The gaseous headspace of pork cutlets was analyzed for volatile cadaverine molecules using either mass spectroscopy or a cantilever sensor featuring a cyclam coating with cadaverine-selective binding properties [220].

**Figure 12:**
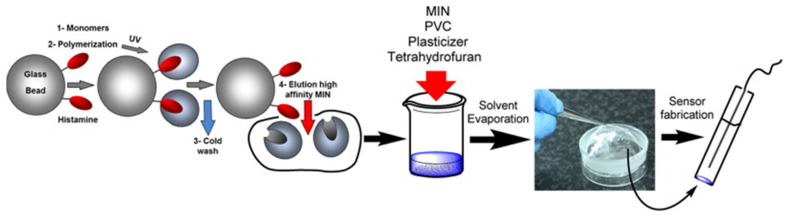
Manufacturing steps of a histamine-sensitive potentiometric electrode [207]. The histamine templates are immobilized on glass beads and the MIP matrix is polymerized under UV irradiation. Through stringent washing steps, one obtains molecularly imprinted nanoparticles (MINs) with high target affinity. The MINs are embedded in a PVC membrane, which serves as the endcap of an electrode filled with histamine solution of known concentration. The sensing principle is based on the intrinsic charge of histamine molecules, and, at slightly acidic pH values, the sensitivity corresponds exactly to the Nernstian limit for divalent ions. The figure is reproduced from ref. [207], Copyright 2014, with permission from Elsevier.

While potentiometric BA detection is rather scarce, Basozabal et al. developed a histamine sensor in which a PVC membrane with embedded molecularly imprinted nanoparticles (MINs) served as histamine-selective element [207]. The MINs, based on methacrylic acid, were synthesized by solid-phase imprinting with the templates fixated on glass beads. and a purification step was applied to retain only the MINs fraction with highest affinity. Figure 12 illustrates the subsequent fabrication steps of the histamine-sensitive membrane. The potentiometric electrode with an inner filling of 1 mM histamine dihydrochloride had a sensitivity of 29.9 mV per decade of histamine concentration, a LoD of 1.1 μM, and a useful concentration range up to 10^−2^ M. Noteworthy, the sensitivity towards histamine was maximal and the cross-selectivity to competitors minimal at pH 3–5, where histamine is double-protonated. When analyzing fish extracts with known histamine contents and histamine-spiked wine (both samples diluted in acetate buffer at pH 5), the measured and real concentrations agreed perfectly with a response time of only 20 s. The approach requires, in addition to the histamine electrode, only a millivoltmeter and a reference electrode. Regarding cyclic voltammetry, Akhoundian and coworkers reported a LoD of 74 pM histamine in 0.1 M KCl solution with hexacyanoferrate as the redox mediator [192]. The working electrode was coated with carbon paste containing finely ground MIP powder, adding increasing histamine concentrations caused a systematic decrease in the peak current amplitudes. The dose–response curve shows two linear regimes with different sensitivities, spanning an analytical range from 0.1 nM up to 400 nM. One may assume that two different mechanisms are involved in transducing the molecular recognition into the output signal and the response, e.g., histidine was not negligible. When adding histamine-spiked human blood serum to the KCl/K_3_[Fe(CN)_6_] solution, the spiked concentrations from 0.5 to 200 nM, were rendered correctly with 95–105% recovery.

MIPs have also been synthesized for the detection of tyramine: Atta and Abdel-Mageed combined molecular imprinting with a sol–gel process to create tyramine-sensitive glassy carbon electrodes [221]. The functional groups of the template were incorporated into a gel that covers the electrode and a higher affinity towards tyramine was established as compared to the competitors dopamine and norepinephrine. Huang et al. fabricated a sol–gel-based MIP layer for tyramine on glassy carbon electrodes: A layer of conductive nanocomposites (nanocarbons combined with gold nanoparticles) was used between the electrode and the imprinted layer to enhance the electrochemical signal [184]. The sensor was used to analyze spiked yoghurt at concentrations of 1–9 μΜ and achieved a tyramine recovery of 90 to 100%. Similarly, Xing and coworkers fabricated an electrochemical tryptamine sensor based on a modified glassy carbon electrode coated with a combination of polypyrrole-sulfonated graphene, multiwalled carbon nanotubes (MW-CNTs), and tryptamine–MIPs. The graphene and MW-CNTs served as electrical conductors, improving the detection signal [195]. This strategy can be modified in several ways, as, e.g., with the example of screen-printed carbon electrodes carrying a layer of the conductive polymer PEDOT:PSS (poly(3,4-ethylenedioxythiophene) polystyrene sulfonate) and a top layer made of MIP-functionalized gold nanoparticles [210].

### 4.5. MIP-Based BA Sensors with Optical Transducers

Histamine–MIPs have been combined with a variety of optical transducers such as fluorescence spectrometry and surface-plasmon resonance (SPR) [189,222]. A recent review by Wu and coworkers covers the topic of fluorescence-based sensors for the detection of histamine in food samples for a plurality of recognition elements [223]. Already, in 2002, Tong et al. synthesized fluorescent histamine–MIPs based on the fluorescent monomer zinc(II)–protoporphyrin. Bound histamine acts as a fluorescence quencher, causing a concentration dependent decay of the fluorescence intensity within an analytically useful range from 0.1 to 1.0 mM [222]. Regarding surface-plasmon resonance (SPR), Jiang et al. synthesized histamine MIPs by bulk polymerization of MAA monomers and EGDM (ethylene glycol dimethacrylate) as a crosslinker directly on the gold surface of SPR chips [189]. The SPR signal, i.e., the shift of the resonance angle, responded linearly to histamine concentrations in buffer from 25 μg/L to 1000 μg/L, corresponding to 225 nM–9 μM. The concept rendered spiked histamine concentrations in carp samples correctly, and the maximum sensitivity was reached at pH 8, in agreement with the theoretical prediction shown in Figure 9a. Gao et al. integrated MIPs-based separation of histamine and AuNPs for signal amplification in SERS (surface-enhanced Raman spectroscopy) in a two-step assay [206]: First, histamine MIPs were incorporated into a PVC film, which served to separate histamine from a spiked matrix of tuna extract. After washing with EtOH, the PVC layer was transferred to a colloidal AuNPs solutions. By changing the ionic strength, histamine released from the MIP and assembled at the surface of the gold nanoparticles. Then, droplets of the supernatant were dried on an aluminum foil, allowing to collect the Raman signature of histamine, which has two characteristic SERS bands at 1304 and 1576 cm^−1^. The peak intensity is a direct measure of the histamine concentration, and it is possible to correct the spectra for the signal of competitors such as histidine. Using a fluorescence-based assay, Haran et al. developed a strategy for the detection of tyramine [224]: CdSe–ZnS core–shell quantum dots were mixed with tyramine and MAA as a functional monomer in an organic solvent together with a crosslinker and an initiator. Molecularly imprinted composite microspheres were obtained, which could successfully indicate the presence of tyramine through the fluorescence-quenching of bound tyramine on the quantum dots.

### 4.6. MIP-Based BA Sensors Based on the Heat Transfer Method HTM

**Figure 13:**
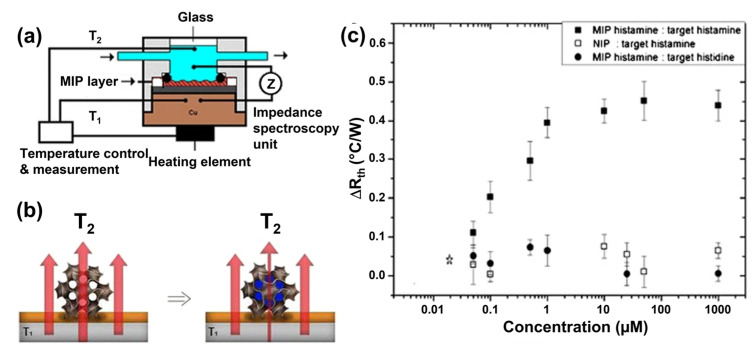
(**a**) Concept of the heat transfer method HTM. A chip, functionalized with a MIP layer, is heated from its backside to a given temperature *T*_1_, e.g., 37 °C. The temperature in the sample compartment, *T*_2_, is measured and allows calculating the heat transfer resistance *R_th_* of the interface. (**b**) The porous structure of the MIP particles (symbolized by an open hexagram) enables facile heat transfer to the supernatant liquid, which diminishes when bound target molecules block the pores, resulting in a drop of *T*_2_ and an increase of *R_th_*. (**c**) Concentration-dependent increase of *R_th_* for increasing histamine concentrations, and the LoD is 100 nM. There is no response to histidine and no histamine response for nonimprinted polymer particles. Figure reprinted with permission by Springer from ref. [194], published in *Analytical and Bioanalytical Chemistry*, Copyright 2013.

Although not well known, histamine can be detected quantitatively with thermometers when using the heat transfer method HTM (see Figure 13a). The method relies on the fact that molecular-scale changes at an interface between a solid and a liquid measurably affect the efficiency of heat transfer from the solid to the liquid [225]. This can be expressed by the thermal resistance *R_th_ =* (*T*_1_ − *T*_2_)/*P* with *T*_1,2_ being the temperatures at the chip backside and within the liquid (measured by thermocouples) and *P* the heating power [226]. In practice, a chip is covered with MIPs in the form of micro-powders as in [194,227], or in the form of graphene–oxide flakes with a MIP shell synthesized by reversible addition fragmentation chain transfer polymerization [228]. Filling up the binding sites by target molecules results in a concentration-dependent increase of *R_th_*, as shown in Figure 13b,c for the case of histamine in buffer. Other molecules that were detected quantitatively using the MIP–HTM combination are nicotine and serotonin; sample volumes can be as little as 2.5 μL, and multiplexing is possible [227]. As an advantage, HTM-based detection can be done on samples with low electrical conductivity, which is hard with electrochemical methods. For the time being, there is still the drawback that HTM requires a temperature-stable environment, which can possibly be solved with the hot-wire technique of ref. [225].

## 5. Biosensors for Biogenic Amines Using Antibodies and Enzymes

### 5.1. Synthesis of Antibodies and Immobilization Strategies

The ability of the antibodies to recognize and bind antigens noncovalently and with high affinity (*K_A_* typically above 10^6^ M^−1^) has turned them into versatile tools for bio-detection, especially in the context of enzyme-linked immunosorbent assays (ELISAs). Antibodies are amongst the oldest recognition elements used in biosensors and they have been developed for a large variety of targets (antigens) such as cells, viruses, proteins, peptides, toxins, and drug residues. Among the different isotypes of antibodies, the most commonly used in biosensors is IgG (immunoglobulin G) and sensors of this category are commonly addressed as immunosensors, since they are mimicking the immune system. Regarding immunosensors for food analysis, we refer the readers to the classical review by Ricci and coworkers [229]. The production of antibodies is based on natural immune reactions in which plasma cells (B lymphocytes) secrete immunoglobulins (antibodies) as a response to a specific antigen. Typically, their production relies on a series of immunizations of laboratory animals such as rodents with a specific antigen, a process that explains the comparatively high cost of this receptor type. Antigens that do not induce an immune response by themselves can be conjugated with a carrier protein, such as ovalbumin and bovine serum albumin. Both monoclonal and polyclonal antibodies are used as biorecognition elements and polyclonal antibodies can be purified directly from blood serum. Monoclonal antibodies are produced with extraction and culture of B-lymphocytes from the spleen or lymph nodes. To culture B-lymphocytes in vitro, it is necessary to fuse spleen isolated B-lymphocytes with immortal myeloma cells. Monoclonal antibodies are then isolated from the supernatant of the hybridoma-cell cultures. An up-to-date review on monoclonal antibody technology can be found in ref. [230], a direct comparison between mono- and polyclonal antibodies is given in ref. [231].

Another way to generate antibodies is through recombinant DNA technology: Recombinant antibodies are genetically engineered proteins, which are selected from antibody gene libraries through phage or cell display technology [232]. Recombinant antibody technology is a fast and controllable process, which does not require the immunization of laboratory animals. Additionally, fragments of antibodies, such as antigen-binding fragments (*Fab*) and single-chain variable fragments (*scFv*), can be advantageous for biosensors due to their small size [233]. The first monoclonal antibodies for histamine detection were reported already in 1986 by Guesdon et al., and, according to ELISA testing, there was no measurable cross-selectivity towards histidine or 1-methyl histamine [234]. Today, monoclonal and polyclonal antibodies for histamine are readily available from several commercial suppliers. For literally all other biogenic amines shown in Figure 1 of this review, mostly polyclonal antibodies are available from commercial sources and used in practice mainly for ELISA tests.

The key advantage of antibodies is their high affinity and selectivity, making them still the preferred choice for analytical laboratories. Notwithstanding, MIPs and aptamers have emerged as valid alternatives, and one has to note that the stability and functionality of antibodies depend strongly on the physicochemical properties of the surrounding medium, such as pH, ionic strength, and temperature. This poses also constraints regarding the coupling chemistry for immobilizing IgG receptors on transducer structures and limitations in sensor regeneration after binding of molecular targets. Physical adsorption is the easiest and least invasive way to attach antibodies to sensor surfaces, where they bind noncovalently through ionic, electrostatic, and hydrophobic interactions in a mix of random orientations. This is widely used on polystyrene surfaces such as well plates in ELISA assays and was also demonstrated for the conjugated (electrically conducting) polymer polyphenylene vinylene [235]. While this is sufficient for a single-shot analysis, the lack of control on the spatial orientation of the antibodies is suboptimal in the sense that the binding sites of IgGs (the *Fab* sections) may not be accessible for capturing antigens. Wiseman and Frank studied the absorption mechanism and orientation of antibodies on hydrophobic surfaces in real-time using a quartz crystal microbalance with dissipation monitoring [236]: At low areal density, antibodies lay in horizontal, flat-on orientation on the surface, and, upon accumulation to higher density, the antibodies reorientate to a mixture of vertical orientations. Chemical binding of antibodies to sensor surfaces can induce an upward orientation of IgGs (binding sites available) together with better resilience against mechanical forces due to washing steps. This was demonstrated recently by Adesina and Mashazi, using glycosylated antibodies against CRP (complement reactive protein) and gold electrodes with a monolayer of boronic acid [237]. For further reading on random and oriented antibody immobilization strategies, comprehensive reviews are available [238,239]. Specifically for biogenic amines, Mutlu and coworkers developed a protocol to bind antihistamine antibodies, in random orientation, chemically to gold-coated quartz crystals [240]: First, the crystals underwent a plasma treatment to obtain a polymeric base layer featuring amino thiols; then, the antibodies were coupled to the thiols using glutaraldehyde as a homo-bifunctional cross-linker.

### 5.2. Immunosensors for Biogenic-Amine Detection

Generally, it is not straightforward to detect BAs with IgG-type receptors, since the receptors have a considerably higher molecular weight and size than their targets. Therefore, a variety of signal amplification strategies come into play. In a first example for a direct assay, Delle and coworkers detected histamine molecules that were linked to bovine serum albumin (BSA), a protein that has half the molecular mass of IgG molecules [241]. Polyclonal antihistamine antibodies were adsorbed physically in random orientation on interdigital electrodes made of conducting reduced graphene oxide rGO. From the impedance signal at low frequencies, they found a concentration-dependent impedance increase in an analytically useful range from 0.1 to 1.0 μM. The sensing effect was attributed to an increase of the charge-transfer resistance *R_ct_* upon binding the histamine–BSA complexes. In a reference test with a SPR platform, the same type of antibodies was immobilized on commercial SPR chips by using a linker layer of protein A that binds IgG molecules at their *Fc* section, thus allowing the *Fab* segments to bind their targets. The SPR sensor achieved the same analytical range, but the relative signal increased faster for low concentrations due to the orientation of the receptors. Key data on this and other immunosensors for BAs are summarized in Table 4, including also sensors that are not explicitly addressed in the text.

Using another amplification strategy, Shkodra et al. published in 2020 a competitive ELISA assay for histamine with a chronoamperometric readout [242]. Antihistamine antibodies were adsorbed on SWCNT-coated silver electrodes that had been screen-printed on flexible PET foils, and the reference and counter electrodes were also printed on the same foil. The electrodes were exposed to histamine-spiked PBS buffer or fish extract, together with a known amount of histamine molecules linked to horseradish peroxidase (HRP). High histamine concentrations in the sample prevent the histamine–HRP complexes from binding to the electrode, and the opposite is true for low histamine concentrations. After washing and incubation with TBM (tetramethylbenzidine) and other chemicals, HRP underwent chemical oxidation and reduction steps that generated the amperometric signal, which was inversely proportional to the histamine concentration in the original sample. The sensor response was linear with respect to the *log* of the histamine concentration from 5 × 10^−3^ to 50 ng/mL (45 pM–450 nM). The response to cadaverine, putrescine, and tyramine was negligible and the sensor rendered spiked histamine concentrations (0.5–50 ng/mL) in filtered fish samples correctly. The assay has a certain complexity and takes several hours, but the measured LoD is remarkably low. Using a conceptually similar approach, Mattson and coworkers developed a histamine assay with colorimetric readout [243,244]: First, histamine was immobilized on sensor chips using a carrier protein. Then, the chips were exposed to samples with spiked histamine concentrations and known amounts of antihistamine antibodies, competing for histamine molecules in solution and immobilized on the chip. After washing, the chips were incubated with a solution of anti-IgG antibodies labeled with carbon-black nanoparticles (alternatively with AuNPs or fluorophores) generating the colorimetric signal.

**Figure 14:**
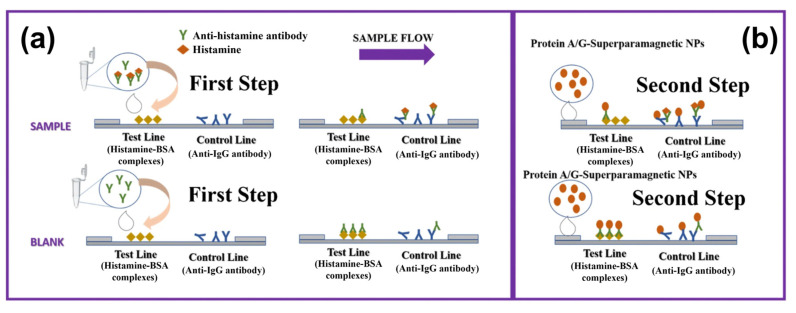
Illustration of the lateral-flow immunoassay in dipstick format developed by Moyano et al. for histamine quantification in red wine samples [245]. (**a**) In the first step, the sample is mixed with a given amount of antihistamine antibodies: Free antibodies are captured at the test line coated with histamine–BSA complexes, histamine–antibody complexes proceed to the control line, where they bind to anti-IgG antibodies. (**b**) In the second step, all antibodies are labeled by gold- or magnetite nanoparticles tethered to the antibodies via the A/G protein. The signal, measured by impedance electrodes underneath the test line, is inversely proportional to the histamine contents of the original sample. Figure reprinted with permission by Springer from ref. [245], published in *Analytical and Bioanalytical Chemistry*, Copyright 2019.

For histamine detection in wine, Moyano et al. developed a lateral-flow immunoassay (LFIA) in dipstick format that resembles a classical antigen test with the difference that target binding and readout are done in two subsequent steps [245]. Nitrocellulose strips were impregnated with histamine–BSA complexes for the test line and anti-IgG antibodies for the control line (see Figure 14). Samples with spiked histamine concentrations were mixed with monoclonal antihistamine antibodies and pulled by capillary forces along the test strip: Free histamine antibodies were captured at the test line and antibody–histamine complexes moved further to the control line and bound by the anti-IgG antibodies. This is again a competitive approach: For a low histamine concentration in the sample, the histamine antibodies assembled mostly at the test line, for a high histamine concentration they accumulated mainly at the control line. In the development step, all antibodies were labeled with either protein A/G functionalized AuNPs for optical visualization or protein A/G functionalized magnetite particles, allowing for impedimetric detection with electrodes underneath the test line. Both transducers gave a similar LoD with 1.2–1.5 mg/L of histamine. Applied to red wine samples in different stages of fermentation and aging, the assay with magnetite beads for electronic detection gave an excellent agreement with reference analyses by ultra-HPLC.

In a final example, Ye et al. utilized superparamagnetic nanoparticles (MNPs of Fe_3_O_4_, 10 nm diameter) to raise the concentration of histamine target molecules and to enhance the impedimetric detection signal [246]. After functionalization with antihistamine antibodies by silane coupling, the MNPs bound histamine present in the sample. Using a magnet, the MNP–histamine complexes were confined in a small liquid volume and the supernatant was discarded. The measurement step was performed in a two-electrode impedimetric cell, with the electrodes being separated by a thin nanoporous alumina membrane featuring 100 nm-wide channels. The membrane and its channels were functionalized by antihistamine antibodies as well, allowing to bind the histamine-loaded MNPs. This results in pore blocking and an impedance increase that is linear in the *log* of the original histamine concentration with a reported LoD of 3 nM. Using this method, Ye et al. also studied the evolution of histamine concentration in saury fish that had been frozen for different durations. The idea of pore blocking resembles the principle of thermometric histamine detection reported in ref. [194] and illustrated in Figure 13b.

**Table 4:** Overview of selected immunosensors for histamine detection based on mono- and polyclonal antibodies together with a brief description of the assay and sensor architecture. To best of our knowledge, there are no publications on immunosensors for BAs other than histamine.

Biogenic Amine	Sample	Sensor Architecture	Transducer	LoD	Analytical Range	Reference
Histamine	Buffer	Carbon nanoparticles-based colorimetric competitive immunoassays on a microarray format	Colorimetric	8 µg/mL	15–101 µg/mL	[243]
	Spiked fish samples	Competitive immunoassay using hydroquinone as electron mediator to measure the catalytic reaction between HRP and H_2_O_2_.	Amperometric	1.25 pg/mL	0.01–100 µg/mL	[247]
	Cod fish, red wine, yogurt	Nanozyme-mediated ratiometric fluorescence immunoassay using histamine-specific antibody labeled with Prussian blue nanoparticles	Fluorescence based on carbon dots	1.2 ng/mL	1.6 ng/mL– 125 µg/mL	[248]
	Spiked PBS buffer	Impedimetric and SPR-based immunosensors using reduced graphene oxide to measure binding of histamine-BSA conjugate	Impedimetric, SPR	0.1 µM	0.1–1 µM	[241]
	Spiked PBS, spiked fish samples	Screen-printed competitive immunosensor based on a silver electrode coated with single-walled carbon nanotubes	Chronoampero-metic	2.48 pg/mL	0.005–50 ng/mL	[242]
	Red wine	Lateral flow colorimetric and magnetic immunoassay using superparamagnetic particles (gold and iron oxide) labels	Magnetic, colorimetric	1.2 mg/L, 1.5 mg/L	1–100 mg/L	[245]
	Saury fish	Nanoporous alumina membrane-based biosensor using biofunctionalized magnetic nanoparticles conjugated with antihistamine antibody	Impedimetric	3 nM	1 µM–40 mM	[246]

### 5.3. Enzymatic Detection of Biogenic Amines

Enzyme-based biosensors for the detection of BAs have been reported extensively (see Table 5 for an overview), and they can be categorized in two groups: (i) sensors developed for the detection of the total amount of BAs and (ii) selective sensors for the detection of specific BAs. To determine the total amount of biogenic amines, one can either use an enzyme with low specificity for the substrate or a system with more than one enzyme. The major family of enzymes suitable for electrochemical biosensors are amine oxidases, which catalyze the oxidative deamination of monoamines, diamines and polyamines into aldehydes, ammonia and hydrogen peroxide. Based on the co-factor, amine oxidases come in subfamilies including the copper-containing diamine oxidases DAO, the monoamine oxidases MAO, and polyamine oxidases PAO [86]. Depending on the targeted analyte, the corresponding enzyme is used. It is worth mentioning that many enzymes have a wide substrate specificity, making it difficult to develop a biosensor that is selective for a specific compound. Already in 1952, Kenten and Mann extracted an amine-oxidizing enzyme from pea seedlings (*Pisum sativum*) that was found to oxidize a wide range of polyamine substrates including histamine, spermine, spermidine, agmatine and hexamethylene-diamine [249]. Using a purification step, it was later shown that the enzyme has an especially high oxidative deamination activity for putrescine and cadaverine [250]. Today, it is widely known as PSAO (pea seedlings amine oxidase) and the first electrochemical biosensor for BA detection using PSAO was reported by Wimmerová and Macholán in 1999 [251], triggering PSAO-related follow-up work in ref. [252]. Since then, enzyme-based BA sensor are a highly flourishing topic and we limit ourselves to the basic principles: In the very recent literature, Verma et al., as well as Ahangari and coauthors, have published fully up to date, comprehensive reviews that address the most recent evolutions of enzymatic BA detection [85,253]. A review dedicated to food spoilage by BAs and bacteria was published by Torre et al., focusing on sensors utilizing screen-printed electrodes [254].

**Figure 15:**
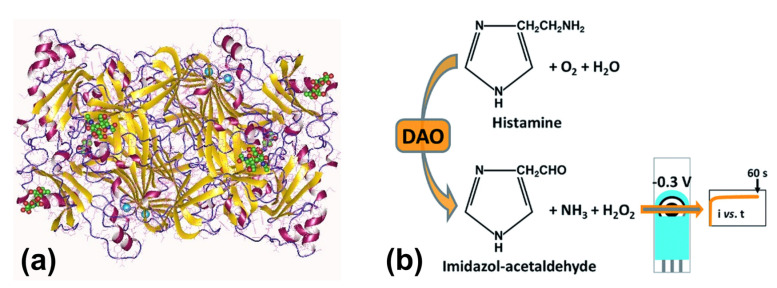
Working principle of enzymatic histamine detection. (**a**) Molecular structure of the DAO enzyme (human variant), the dimeric molecule has a total weight of 170 kDa [259]. The four calcium ions of the dimer are presented in light blue, the two copper ions in orange, and the 10 n-acetylglucosamine groups in green-red. (**b**) Enzymatic conversion of histamine to imidazole-acetaldehyde with NH_3_ and hydrogen peroxide as additional educts. The electrochemical reduction of H_2_O_2_ to water causes a cathodic current that is linearly proportional to the histamine concentration in the sample. (**b**) is adopted from ref. [256], by permission of the Royal Society of Chemistry, Copyright 2019.

A widely used enzyme for histamine detection is diamine oxidase DAO, previously known as histaminase or amine oxidase, copper containing, 1. Figure 15a shows the molecular structure of human DAO that can, e.g., be found in the intestinal mucosa; DAO is a dimer consisting of two subunits with a molecular weight of 85 kDa each and the enzymatic activity is attributed to one copper atom per subunit [255]. The deamination reaction can be written as R-CH_2_NH_2_ + O_2_ + H_2_O → R-CHO + H_2_O_2_ + NH_3_, which is illustrated for the specific case of histamine in Figure 15b. Torre and coworkers developed an especially fast chronoamperometric histamine sensor based on DAO (extracted from porcine kidney) that was immobilized together with bovine serum albumin BSA and glutaraldehyde for crosslinking on a working electrode made by screen-printed carbon ink [256]. DAO catalyzes the deamination of histamine in presence of oxygen and water to imidazole-acetaldehyde with ammonia and hydrogen peroxide as additional educts. Using a counter- and a pseudo-reference electrode on the same chip, H_2_O_2_ undergoes a cathodic reduction reaction according to the scheme H_2_O_2_ + 2 e^−^ + 2 H^+^ → 2 H_2_O. The current output at a given voltage (−0.3 V) was already stable within less than 60 s and the current amplitude was linear with respect to spiked histamine concentrations from 1–100 mg/L; total sample volumes of only 40 μL were sufficient for the analysis. Furthermore, spiked histamine concentrations in fish-extract samples (hake and mackerel) were determined correctly for absolute concentrations from 9 μM up to 4 mM. During extraction of the fish, the samples were actually boiled for 20 min, which is considered uncritical due to the high thermal stability of histamine. As a side note, the assay should be performed at a close to neutral pH and there was a nonselective response towards spermine, spermidine, and phenylethylamine. The work by Torre et al. also contains references suggesting that the deamination reaction can be catalyzed by metallic copper, Cu_3_(PO_4_)_2_, and rhenium (IV) oxide, which may be considered as cheaper alternatives to DAO. In a direct comparison between histamine sensors based on DAO and MAO, Koçoğlu et al. found a roughly 1.5 times higher sensitivity for the DAO enzyme [257]. Vice versa, according to Kaçar and coworkers, MAO performs better than DAO when it comes to cadaverine detection, which was performed on cheese samples and validated by HPLC [258].

Already in 2007, Keow et al. developed an amperometric histamine sensor in which DAO was embedded in a hydrogel coating on a carbon-paste electrode [260]: The sensor had a response time below 1 min and reached a LoD of 0.65 ppm, which is meaningful in the sense that it is far below the legal histamine limits for fish samples. Using this sensor, Keow and coworkers analyzed the histamine concentration in tiger prawns that had been exposed to a temperature of 30 °C for 5 h and found good agreement between the sensor-derived data and HPLC reference results. Pérez and coauthors combined DAO with HRP (horseradish peroxidase), lowering the required potential to −50 mV vs. Ag/AgCl and obtained an LoD of 0.17 μM with a linear range from 0.3 to 20 μM [261]. The sensor gave correct results for histamine in several fish species (sardines, mackerel, greater weever) as checked independently by ELISA testing. To broaden the applicability also towards putrescine and cadaverine, Leonardo and Campàs developed an amperometric sensor array, allowing to move DAO-functionalized magnetic beads towards three different carbon electrodes featuring either a cobalt complex, or Prussian blue or horseradish peroxidase combined with electrochemically active osmium [262]. A sensor featuring DAO and MAO together with HRP allows to quantify the total BA amount in a variety of food samples [263]. For more specific information to determine histamine and putrescine concentrations separately, Henao Escobar et al. developed a dual sensor based on the enzymes histamine dehydrogenase and putrescine oxidase instead of DAO that allows to measure the total BA contents of samples [264,265].

Putrescine oxidase belongs to the monoamine oxidases and catalyzes the oxidation (deamination) of putrescine with higher selectivity than DAO. Bóka et al. developed an amperometric putrescine sensor in which putrescine oxidase and HRP were embedded in a gel, together with a mediator and a cross-linker, on carbon electrodes [266]. The putrescine oxidase had been extracted from the bacterium *Kocuria rosea*. The enzymatic activity was also assessed with respect to other BAs, giving a relative activity (100% for putrescine) of 1.7% for histamine and up to 10.7% for cadaverine. The sensor was used to determine the native putrescine contents in a variety of degassed beer samples, ranging from 1.5 to 15.2 mg/L. While the sensor slightly overestimated the real concentrations, still an excellent correlation was found with HPLC-based reference data. Putrescine- and cadaverine detection has also been reported with a human-type MAO enzyme and selectivity between the two was achieved by different MAO quantities on two carbon electrodes, one of which featuring additionally AuNPs [267]. This way, it was possible to quantify the contents of both BAs in octopus samples. In addition, ref. [267] provides a complete overview on all enzymes that have been utilized for sensor-based BA detection up to the year 2013. To our knowledge, this inventory of enzymes is still valid today. Regarding spermine and spermidine, both are oxidized by polyamine oxidase while spermine oxidase (belonging to the MAO category) decomposes spermine exclusively [268,269]. Boffi and coworkers measured concentration ranges from ca. 4 μM to 4 mM in the blood samples and confirmed their results by gas chromatography-mass spectrometry GC-MS [268]. A recent spermine sensor based on electrochemiluminescence of Au−Ag bimetallic nanoclusters has a stated LoD below 1 pM, without using any enzymes, but to best of our knowledge the result was not yet reproduced independently [270].

As an example of an enzymatic tyramine sensor, we refer to recent work by Erden et al. in which tyrosinase was immobilized on glassy carbon electrodes using carbon nanofibers, AuNPs and additional components [271]. The oxidase tyrosinase is produced by plants and animals, where it converts tyramine to the pigment melanin. Using an amperometric readout scheme, the authors found an analytically useful range from 0.2–48 μM and determined native and spiked tyramine concentrations in soy sauce with recovery factors around 100%. Interestingly, the sensor was highly selective and the response towards seven other biogenic amines was far below 1% of the specific signal for tyramine. Erden and coworkers developed also a tyramine sensor without the specific enzyme tyrosinase, but with MAO and DAO instead [272], the enzyme plasma-amine oxidase can serve for the same purpose [273]. As a second example of a tyrosinase-based sensor, Dalkıran et al. coated screen-printed carbon electrodes with an electropolymerized layer of poly-L-lysine, that was drop-coated with a solution of tyrosinase, chitosan, and Fe_3_O_4_ nanoparticles and finally enclosed with a Nafion foil [274]. The sensor had a LoD of 75 nM, a linear range from 0.49–63 μM, and was utilized to determine the tyramine contents in liquefied and filtered cheese samples. Alternative electrode coatings onto which tyrosine can be physically adsorbed without losing its enzymatic activity are polypyrrole and carboxyl-functionalized carbon nanotubes [275,276]. Furthermore, da Silva and coauthors functionalized glassy carbon electrodes with gold nanoparticles embedded in a layer of poly(8-anilino-1-naphthalene sulphonic acid) onto which tyrosinase was adsorbed. The resulting amperometric tyramine sensor had a linear response range from 10–120 μM, allowing to quantify native and spiked tyramine concentrations in yoghurt, cheese, beer, and red wine [277]. The sensitivity remained stable after storage for several weeks. According to a recent work by Gonçalves da Silva, it might even be possible to quantify tyramine in wine by voltammetry due to a characteristic irreversible oxidation peak at +0.64 V without using enzymes [278]. For the time being, it is not yet confirmed whether this straightforward, low-cost method is sufficiently selective towards competing BAs.

### 5.4. Commercially Available Fast Tests for Histamine Detection

Histamine is the only biogenic amine for which fast tests are available from commercial sources (see an overview in Table 5). All tests come as test strips and the result is visualized by a color code or a change in color intensity. While the result is visible after a couple of minutes, the information can be considered as semi-quantitative. Converting enzymatic reactions to a colorimetric response is well established in the literature (see, e.g., ref. [279]). The lowest quantifiable concentration is 10 ppm for the HistaSure^TM^ test, which corresponds to a rather high concentration of ca. 550 μM in an aqueous matrix. Nevertheless, these tests allow to determine quickly whether fish samples agree with the European regulations or not (see ref. [63]). In a recent field study on yellowfin tuna marketed in Sardinia/Italy, Pais and coworkers employed the HistaSure^TM^ test in parallel with liquid-chromatography-mass spectrometry [280]. If exact information on concentrations in the ppb range is needed, also ELISA kits are available from several suppliers: Here we leave the idea of on-site testing since ELISA tests require a plate reader and auxiliary chemicals, typically available in analytical laboratories. The performance of several commercially available ELISA kits and immunosensors for histamine detection and quantification are reviewed in a publication by Köse and coworkers [281].

## 6. Summary, Outlook and Conclusions

Biogenic amines are naturally present in many types of food and beverages, but their accumulation in high concentrations, often due to bacterial food spoilage, implies potential health risks for consumers, especially for sensitized persons. The major risk stems from histamine (scombroid food poisoning), which can be found mainly in histidine-rich fish, cheese, and fermented products. Both, the European Commission and the U.S. Food and Drug Administration have strict regulations in place on maximum histamine amounts in fish and its derived products. High-performance liquid chromatography is the recognized and recommended analytical reference method, which is evidently bound to analytical laboratories. Hence, there is a gap that can be filled by fast, user-friendly, and low-cost testing devices: If they detect anomalously high BA concentrations in a certain food product, refined laboratory testing can be done in a second step with high selectivity and extremely low limits of detection.

Bio- and biomimetic sensors are promising tools for the on-site detection of BAs during food processing because they can facilitate the quality control of food and beverages in several ways: By testing on-site, directly within a production environment instead of an analytical laboratory, and by testing more frequently. This implies certain requirements regarding the cost per analysis and the user-friendliness of the device. The sensors developed until date for this purpose use either natural bio-recognition elements (antibodies and enzymes), aptamers selected from nucleic acid libraries, or synthetic receptors in the form of molecularly imprinted polymers (MIPs). The existing, commercially available fast tests make in all cases use of antibodies or enzyme technology, which are at a mature technology level. While these sensors do not reach detection limits at the nanomolar scale, they are user friendly and allow for a simple but efficient readout by visual inspection. Receptor-free sensors, based on the electrochemical fingerprints of biogenic amines, exist as well; however, they do not yet reach the degree of selectivity and low detection limits of the affinity-based concepts.

Biogenic amine sensors are generally meant for a one-time analysis and repetitive or even continuous sensing over extended time scales are beyond the capabilities of current technology. Nevertheless, this might be of interest for the food industry and whether this is possible depends mainly on the type of receptors. In principle, all biosensors should be able to render concentrations that are increasing as a function of time, as long as the upper limits of detection have not been reached. For monitoring decreasing concentrations, the situation is different: Affinity-based receptors (MIPs, aptamers, antibodies) are optimized regarding their target affinity, meaning they would respond to a decreasing concentration only with considerable delay. Still, it is possible to “reset” and regenerate the sensors by breaking the bonds between targets and receptors by chemical or physicochemical means. For enzyme-based sensors, and those without a receptor element, there is no fundamental reason why they should not be able to monitor a decrease in BA concentration correctly. However, when analyzing complex food matrices, fouling effects take place and a regular cleaning is still indispensable.

Regarding the transducer principles, all reviewed concepts have proven successful in BA detection, including the electronic, electrochemical, optical, gravimetric and thermal generation of quantifiable signals. Irrespective of the employed receptor type, most sensors discussed within this review reach at least micro- or nanomolar limits of detection, which holds also for complex matrices. These detection limits are generally low enough for food safety applications and there is considerable knowledge of preparing liquid extracts from solid food samples. Based on the number of recent publications, MIPs and aptamers gained enormously in popularity: This is certainly related to the fact that their traditional shortcomings in terms of affinity and selectivity seem to be resolved and both receptor types become increasingly available from commercial suppliers. While aptamers were discovered already in 1990, it is only since a few years that aptamers were developed for BAs, including histamine. Hence, one can expect that this research line will gain considerable popularity in future. Of course, the prize of these innovative receptor types needs to further come down and variability between batches needs to be addressed and reduced. For the case of MIPs, we see electro-polymerization as a promising synthesis route due to its comparatively low cost and potential for upscaling to serial production.

Keeping the idea of on-site detection in mind, especially the electronic and electrochemical sensing platforms lend themselves for miniaturization and integration in portable, pocketsize devices. Ultimately, electronic tests for histamine and other BAs may come with a design that is as “simple and straightforward” as glucose meters for diabetes patients. There is a still a way to go and BA concentrations in spoiled food are considerably lower than glucose in the blood, but all basic requirements (receptors, sample preparation, readout instrumentation) are available for integration in compact “BA meters”. These hypothetic BA meters do not need to beat the sensitivity and selectivity of mass spectrometry, but they will be a step ahead in comparison to fast tests with colorimetric readout by eye: They will provide absolute concentrations and span a considerably broader range of quantifiable concentrations. Potential users in first place might be food-producing companies, persons with an allergic disposition, and patients under enzyme-inhibiting treatments. As of today, there are already food companies emphasizing the low (or zero) histamine contents in their products, which creates confidence and awareness at the side of consumers.

## Figures and Tables

**Table 1:** Overview of aptamers developed for the recognition of a variety of biogenic amines together with their selection method, type and base number of the nucleic acid, and the dissociation constant *K_D_*. Low *K_D_* values correspond to a high level of selectivity. The references refer to the original work in which a given aptamer was reported for the first time, irrespective of follow-up work based on the earlier selected nucleotide sequences.

Biogenic Amine	Selection Route	Nucleic Acid	*K_D_* (mol/L)	Reference
Histamine	SELEX	DNA, 49 bases	72.8 ± 13.9 × 10^−9^	[131]
Histamine	SELEX	DNA, 99 bases	3.08 ± 1.13 × 10^−9^	[132]
Histamine	Separate-SELEX	DNA, 80 bases	11.62 ± 3.24 × 10^−9^	[122]
Tryptamine			6.30 ± 1.76 × 10^−9^	
Tyramine	SELEX	DNA, 38 bases	0.2 ± 0.4 × 10^−6^	[127]
Spermine	Capture-SELEX	DNA, 40 bases	9.65 ± 0.90 × 10^−9^	[133]
Ethanolamine	SELEX	DNA, 96 bases	6 ± 3 × 10^−9^	[128]
Dopamine	SELEX	RNA, 57 bases	1.6 × 10^−6^	[134]

**Table 3:** Overview of selected BA sensors using molecularly imprinted polymers as selective recognition elements. While the table is not exhaustive, it is evident that MIPs are highly versatile, and the limits of detection are remarkably lower than in the case of the commercially available fast tests for histamine discussed in Section 5.4. While A summarizes the sensors for histamine detection, B reviews the publications on sensors for various other biogenic amines.

A	Sample	Sensor Architecture	Transducer	LOD	Analytical Range	Reference
	Spiked fish products	Hydrolytic cross-linking of silanol groups using a sol–gel process	QCM	7.49 × 10^−4^ mg/kg	0.11 × 10^−2^– 4.45 × 10^−2^ mg/L	[203]
	Canned tuna	Bulk polymerized MAA particles, OC_1_C_10_-PVV and PVC adhesive	Impedimetric QCM	Below 1 nM 1 µM	0–12 nM 1–100 µM	[183]
	PBS buffer solution	Bulk polymerized MAA particles, OC_1_C_10_-PVV adhesive	Impedimetric	2 nM	3–12 nM	[193]
	PBS buffer solution	Bulk polymerized MAA particles, OC_1_C_10_-PVV adhesive	Impedimetric	15 nM	500–1000 nM	[197]
	PBS + KCl solution	Electro-polymerization of Pyrrole on B:NCD diamond film	Impedimetric	Not mentioned	Not mentioned	[204]
	Intestinal fluids	Electro-polymerization of Pyrrole on Titanium wires	Impedimetric	1 nM	1 nM–10 µM	[197]
Histamine	Spiked buffer	Bulk polymerized MAA deposited on a PDMS stamp.	Admittance spectroscopy	50 nM	50 nM–1000 nM	[205]
	Histamine buffer solution	Bulk polymerized MAA particles, OC_1_C_10_-PVV adhesive	Heat transfer resistance HTM	30 nM	0.2 µM–1 µM	[194]
	PBS solutions with K_3_[Fe(CN)_6_]	Bulk polymerization of MAA on CP electrodes	Voltammetry	7.4 × 10^−5^ µM	10^−4^–10^−3^ µM, 7 × 10^−3^–4 × 10^−1^ µM	[192]
	Canned tuna	Bulk polymerized MAA, MIPs-PVC film combined with AuNPs	SERS	Not mentioned	Not mentioned	[206]
	Carp fish	Bulk polymerized MAA on a thin-film gold SPR sensor chip	SPR	24.9 µg/L	25–1000 μg/L	[189]
	Wine and fish matrices	Solid-phase imprinted MAA, MINs in PVC membrane	SPR, potentiometric	1.1 µM	1 µM–10 mM	[207]
	Purified fish extract	Precipitation polymerization of MAA	Competitive fluoro-immunoassays, BODIPY C_9_H_7_BN_2_F_2_	1 µM	1–430 µM	[208]
**B**	**Sample**	**Sensor architecture**	**Transducer**	**LOD**	**Analytical** **range**	**Reference**
Tryptamine	Cheese, lacto-bacillus beverage	MIP electropolymerized on conductive, MWCNT modified GCE	Amperometric	42 nM	60 nM – 30 μM	[185]
	Meat samples	MIP made of MAA and AM polymerization, based on CD-embedded COFs	Fluorimetric, luminescent carbon dots	7 µg/kg	0.025–0.4 mg/kg	[209]
Tyramine	Milk	MIP-based Tyr extraction before detection with AuNP modified SPCE	Voltammetric	0.00231 µM	5–100 nM	[210]
	Yoghurt	MIP by precipitation polymerization on MWCNT/AuNP modified GCE	Voltammetric	0.057 µM	0.108 µM–10 µM	[184]
Spermidine	CCl_4_–spermidine solution	Noncovalent polymerization of MAA	Absorption spectroscopy	0.3 mmol/L	Not mentioned	[211]
	Spiked serum samples	MIP membrane by bulk polymerization of quinolyl-b-CD	Fluorimetric, quinoline C_9_H_7_N	5 × 10^−7^ mol/L	0.5–200 µM	[212]
Putrescine	Putrescine aqueous solution	Bulk polymerization of PVA500 on glass slide and Petry dish	Colorimetric	4 mg/g	4–24 mg/g	[186]
	Putrescine in ethanol	Bulk polymerization of PVA1799, 1,4-butanediol. MIP deposited on NFM	Colorimetric	Not mentioned	Not mentioned	[187]
Histidine	Human urine	AM films on Au electrode by electro-polymerization (cyclic voltammetry)	Amperometric	D-His: 0.01 µM L-His: 0.1 µM	D-His: 100 nM–10 µM L-His: 100 nM–10 µM	[213]
	Spiked water and serum samples	Bulk polymerization of TEOS bound to Fe_3_O_4_ particles	Potentiometric	0.01 µM	0.01 µM–1 µM 30 µM–70 µM	[214]

Abbreviations in A, B: AA: acrylic acid, AM: acrylamide, AuNPs: gold nanoparticles, B:NCD: boron-doped nanocrystalline diamond, CD: carbon dots, COF: covalent organic framework, CP: carbon paste, GCE: glassy carbon electrode, HTM: heat transfer method, MAA: methacrylic acid, MINs: molecularly imprinted nanoparticles, NFM: nanofiber membrane, MWCBNT: multi-walled carbon nanotubes, PBS: phosphate-buffered saline solution, PDMS: polydimethylsiloxaan, PPV: poly-phenylenevinylene, PVA: polyvinylalcohol, PVC: polyvinylchloride, QCM: quartz crystal microbalance, SERS: surface enhanced Raman scattering, SPCE: screen-printed carbon electrode, SPR: surface-plasmon resonance, and TEOS: tetraethoxysilane.

**Table 5:** Overview with examples of commercially available fast tests for histamine detection.

Recommend Sample Type	Name of Test	Concentration Range	Recognition Element	Time to Result	Manufacturer
Seafood, wine, juice, milk, dairy	QuantiQuik™	20 ppm–200 ppm	Enzyme: Histamine dehydrogenase	15 min	BioAssay Systems, Hayward, CA, USA
Fish products (fresh, frozen, canned marine products)	HistaSure™ Fish Rapid Test	10 ppm–200 ppm	Immunogold labeled histamine antibody	5 min	BioSystems S.A., Barcelona, Spain
Seafood, fish sauce, fish meal, wine, milk	HistaStrip™	15 ppm–75 ppm	Histamine-specific enzyme	4 min	PerkinElmer Inc., Waltham, MA, USA
Fresh, canned and salted fish, fish in oil, fish meals	AgraQuant^®^	0.5 ppb–50 ppb	Antibody, competitive ELISA	not given	Romer Labs, Tulln an der Donau, Austria

## Data Availability

No new data were created or analyzed in this study. Data sharing is not applicable to this article.
